# A Cytochrome *c*‐Containing Periplasmic Nitrate Reductase in the Acetogen *Sporomusa ovata*

**DOI:** 10.1111/1462-2920.70228

**Published:** 2026-01-07

**Authors:** Lara M. Waschinger, Anja Poehlein, Rolf Daniel, Florian P. Rosenbaum, Volker Müller

**Affiliations:** ^1^ Molecular Microbiology & Bioenergetics Institute of Molecular Biosciences, Johann Wolfgang Goethe University Frankfurt Germany; ^2^ Göttingen Genomics Laboratory, Institute of Microbiology and Genetics Georg August University Göttingen Göttingen Germany

## Abstract

*Sporomusa ovata* is one of the few acetogenic bacteria that have cytochromes and quinones, but their role is unknown. In addition to reducing CO_2_ to acetate, *S. ovata* can also use nitrate as an alternative electron acceptor, but the enzymes involved, a possible function of cytochromes and quinones and the bioenergetics of nitrate reduction remain elusive. Under heterotrophic and autotrophic growth conditions, the presence of nitrate led to higher optical densities and decreased acetate production. Under heterotrophic conditions with fructose as an electron donor, nitrate was preferred over CO_2_ as an electron acceptor. Under autotrophic conditions with H_2_ as an electron donor, nitrate and CO_2_ were used simultaneously. Genome analyses revealed a nitrate reduction island with genes encoding a periplasmic cytochrome *c*‐containing nitrate reductase, nitrite reductase, cytochrome *c* and heme biosynthesis. The expression of this nitrate reduction island was strongly induced by the presence of nitrate. Enzyme assays demonstrated membrane‐bound nitrate and nitrite reductase activities exclusively in nitrate‐grown cells. Heme peroxidase staining confirmed the presence of cytochromes in nitrate‐grown cells. In sum, nitrate reduction to ammonium is catalysed by a membrane‐bound electron transport chain involving cytochromes and potentially coupled to energy conservation in *S. ovata* .

## Graphical abstract

*Sporomusa ovata* , a cytochrome‐containing, acetogenic bacterium, conserves energy by reduction of carbon dioxide to acetate. Nitrate serves as additional electron acceptor and is reduced to ammonium, resulting in increased growth and reduced acetate production. Genome and transcriptome analyses revealed a nitrate reduction island, which is induced by the presence of nitrate. Enzyme measurements and heme peroxidase staining showed a membrane‐bound electron transport chain involving cytochromes for nitrate reduction to ammonium.

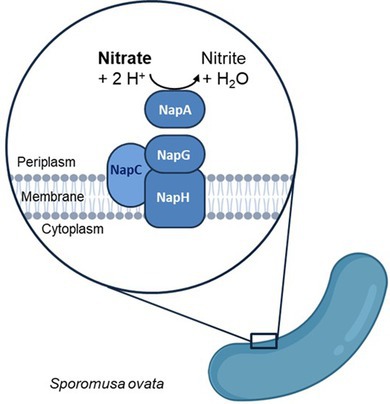

## 1 Introduction

Acetogenic bacteria are a specialised group of strictly anaerobic bacteria characterised by their ability to reduce CO_2_ to acetate. CO_2_ fixation is catalysed by the Wood‐Ljungdahl pathway (WLP), a two‐branched linear pathway in which two molecules of CO_2_ are reduced and condensed with CoA to form acetyl‐CoA, which is subsequently converted to acetyl‐phosphate and then acetate (Drake et al. 2008; Ragsdale and Pierce 2008). CO_2_ is used as an external electron acceptor for electrons derived from the oxidation of sugars, sugar alcohols, alcohols, molecular hydrogen or carbon monoxide (Drake et al. 2008; Wood and Ljungdahl 1991). During chemolithoautotrophic growth on H_2_ + CO_2_ one ATP is consumed for the activation of formate to formyl‐THF and one ATP is gained in the acetate kinase reaction; thus, the net ATP synthesis by substrate‐level phosphorylation is zero. Therefore, additional chemiosmotic mechanisms of ATP synthesis must exist and two enzyme complexes are known that catalyse the generation of a transmembrane electrochemical ion gradient—the Rnf and Ech complex. The electrochemical ion potential across the membrane is then used by an ATP synthase to drive the synthesis of ATP (Schuchmann and Müller 2014).

In the WLP, CO_2_ acts as an external electron acceptor, but some acetogens can use alternative electron acceptors such as nitrate, dimethyl sulfoxide, thiosulfate or acrylates (Drake et al. 1994; Ragsdale and Pierce 2008; Rosenbaum et al. 2022; Seifritz et al. 1993). Whether or not the reduction of the alternative electron acceptor is energy‐conserving depends on the species and the electron acceptor. For example, caffeate reduction in *Acetobacterium woodii* is not directly coupled to membrane‐bound energy conservation (Hess et al. 2011; Imkamp and Müller 2002). However, it is indirectly linked to ATP synthesis. The electron bifurcating caffeyl‐CoA reductase reduces ferredoxin and reoxidation of reduced ferredoxin fuels the respiratory Rnf complex (Biegel and Müller 2010; Imkamp et al. 2007; Imkamp and Müller 2002; Müller et al. 2008). In contrast, reduction of DMSO by cytochrome‐ and quinone‐containing *Moorella thermoacetica* is membrane‐bound and energy‐conserving by a chemiosmotic mechanism (Rosenbaum et al. 2022). In addition to DMSO, the reduction of the alternative electron acceptor nitrate may also be energy‐conserving in cytochrome‐containing acetogens (Fröstl et al. 1996; Seifritz et al. 1993).

In general, not much is known about the role of cytochromes in acetogenic bacteria. It has been known for more than 50 years that there are a few acetogens harbouring cytochromes in addition to the respiratory enzyme complexes Rnf or Ech, but the function of cytochromes has remained unexplored for a long time. Recently, it was shown that *S. ovata* H1^T^ has a cytochrome‐dependent third way of energy conservation (Kremp et al. 2022). In this pathway, a membrane‐bound, periplasmic, cytochrome *b*‐containing hydrogenase serves as electron donor for the membrane‐associated methylene‐THF reductase, an enzyme of the methyl branch of the WLP. The electrons produced by the oxidation of molecular hydrogen are conducted through the membrane via cytochrome *b* and then used to reduce methylene‐THF (Kremp et al. 2022). The resulting H^+^ gradient can be used for ATP synthesis.

Some cytochrome‐containing acetogenic species such as *M. thermoacetica* and *Sporomusa ovata* can reduce nitrate to ammonium (Balk et al. 2010; Seifritz et al. 2002). The genome of *S. ovata* strain An4, closely related to *S. ovata* H1^T^, has nitrate and nitrite reduction genes as well as *c*‐type cytochrome biosynthesis genes (Visser et al. 2016). Proteome analyses of strain An4 showed the presence of the nitrate reductase, nitrite reductase and cytochrome *c* biosynthesis proteins when it was grown on methanol in the presence of nitrate (Visser et al. 2016). Nitrate and nitrite reductase activities were measured in the crude extract of cells grown on methanol in the presence of nitrate (Visser et al. 2016). However, a potential role of the nitrate reductase in energy conservation was not discussed, but a similar scenario as for the methylene‐THF reductase in *S. ovata* is also conceivable for nitrate reduction. In contrast, the cytochrome‐devoid acetogen *Clostridium ljungdahlii* has soluble cytoplasmic nitrate and nitrite reductases and nitrate reduction is not energy‐conserving (Emerson et al. 2019; Klask et al. 2022).

In this study we have shed light on the physiological role of nitrate reduction in *S. ovata* H1^T^ and will provide evidence for membrane‐bound nitrate and nitrite reductase activities. Furthermore, biochemical analyses revealed the presence of cytochromes in cells grown in the presence of nitrate. Our data are in line with the hypothesis that nitrate reduction involves cytochromes and is energy‐conserving in *S. ovata* .

## 2 Experimental Procedures

### 2.1 Cultivation of *S. ovata*

*S. ovata* H1^T^ (DSM 2662^T^) was obtained from the Deutsche Sammlung von Mikroorganismen und Zellkulturen (DSMZ; Braunschweig, Germany), routinely grown under strictly anoxic conditions at 30°C and cultivated in a volume of 5, 50 or 500 mL in modified DSM medium 311. Glycine betaine and Na_2_S × 9 H_2_O were omitted from the media and 0.6 g L^−1^ cysteine‐HCl × H_2_O was used as reducing agent. For CO_2_‐rich conditions the media contained bicarbonate (47.6 mM). As substrates for growth, 20 mM fructose or a gas phase of H_2_ + CO_2_ (80:20 [v/v]) (1 bar overpressure) were used. Growth was monitored by determining the optical density of the culture at 600 nm (OD_600_).

### 2.2 Preparation of Resting Cells

All steps were performed under strictly anoxic conditions in an anoxic chamber (Coy Laboratory Products Inc., Grass Lake, Michigan, USA) filled with N_2_/H_2_ (96%–98%/2%–4% [v/v]). Pre‐cultures of *S. ovata* were incubated in 50 mL media until the late exponential growth phase. The main cultures (500 mL) were inoculated with the respective pre‐cultures (10% of the volume of the main culture). The cells were harvested in the late exponential growth phase. Therefore, the culture was transferred to gas‐tight plastic tubes and centrifuged for 15 min at 8500 rpm and 4°C (JA‐10/Avanti; Beckman Coulter GmbH, Krefeld, Germany). The supernatant was discarded and the cells were then washed in imidazole buffer (50 mM imidazole‐HCl, 20 mM MgSO_4_, 20 mM KCl, 2 mM DTE, 4 μM resazurin, pH 7.0). The cells were sedimented for 15 min at 8500 rpm and 4°C (JA‐25.50/Avanti; Beckman Coulter GmbH, Krefeld, Germany); the supernatant was discarded and the cells were then resuspended in 5 mL imidazole buffer, transferred to a 20 mL Hungate tube and sealed gas‐tight. The residual hydrogen from the anaerobic chamber was removed by changing the gas phase to 100% N_2_. The protein concentration was determined as described before (Schmidt et al. 1963) and the resting cells were stored at 4°C until use.

### 2.3 Cell Suspension Experiments

Serum flasks with a total volume of 120 mL were sealed gas‐tight with butyl rubber stoppers and gassed with N_2_/CO_2_ (80:20 [v/v]) or N_2_ for 20 min to set the required gas atmosphere for the subsequent experiments and to remove oxygen from the serum flasks. The cells were resuspended in 10 mL of imidazole buffer (50 mM imidazole‐HCl, 20 mM MgSO_4_, 20 mM KCl, 2 mM DTE, 4 μM resazurin, pH 7.0) in the serum flasks to a final protein concentration of 1.5 mg m L^−1^. If required, 60 mM KHCO_3_ was added to the buffer. The resting cells were pre‐incubated at 30°C in a water bath (150 rpm). To start the experiment, 20 mM fructose was added as substrate; for experiments with H_2_ + CO_2_ the gas phase was changed to H_2_ + CO_2_ (80:20 [v/v]) (1 bar overpressure), respectively. During the experiments, 1 mL samples were taken for metabolite analyses.

### 2.4 Analytical Methods

The fructose, acetate, nitrate, lactate and ethanol concentrations were analysed by high‐performance liquid chromatography (HPLC) (1260 Infinity II LC System; 1260 Infinity II Quaternary Pump, 1260 Infinity II Vial sampler, 1260 Infinity II Multicolumn Thermostat, 1260 Infinity II Diode Array Detector, 1260 Infinity II Refractive Index Detector; Agilent Technologies, Santa Clara, California, USA). Therefore, samples were centrifuged for 5 min at 13300 rpm (Thermo Scientific Heraeus Fresco 17; Fisher Scientific GmbH, Schwerte, Germany). Sample volumes of 200 μL of the supernatant were transferred via syringe filters (4 mm Millex‐LH Syringe Filters; Merck KGaA, Darmstadt, Germany) into 400 μL flat‐bottom glass inlets that fitted into 2 mL glass vials (Agilent Technologies, Santa Clara, California, USA). The glass vials were sealed with screw caps. For each sample, 5 μL were injected by the autosampler. As mobile phase, 5 mM degassed and filtered sulphuric acid (H_2_SO_4_) was used at a flow rate of 0.6 mL min^−1^. The components of the sample were separated using a Hi‐Plex H 300 × 7.7 mm column with its precolumn Hi‐Plex H Guard 50 × 7.7 mm (Agilent Technologies, Santa Clara, California, USA). The sample components were analysed with a refractive index detector at 55°C and a diode array detector operating in the range of 200–220 nm. The reference cell of the refractive index detector was purged with 5 mM H_2_SO_4_ before analysis and the run time of one sample analysis was 30 min. The concentration of H_2_ was determined by using a gas chromatograph (7890B GC System; Agilent Technologies, Santa Clara, California, USA). A sample volume of 70 μL was injected at 100°C and separated on a ShinCarbon ST80/100 column (2 m × 0.53 mm; Restek Corporation, Bellefonte, Pennsylvania, USA). Nitrogen was used as carrier gas with a head pressure of 400 kPa and a split flow of 30 mL s^−1^. The oven was kept at 40°C and the samples were analysed with a thermal conductivity detector at 100°C. The nitrite and ammonium concentrations were determined using the Nitrite/Nitrate Assay Kit (Cat. No. 23479; Sigma) (Merck KGaA, Darmstadt, Germany) and the Ammonia Assay Kit (Cat. No. AA0100; Sigma) (Merck KGaA, Darmstadt, Germany), respectively.

### 2.5 Transcriptome Analyses

For transcriptome analyses, cells were grown on 20 mM fructose or 20 mM fructose + 20 mM nitrate and harvested in the exponential growth phase with OD_600_ of 0.4 and 0.46, respectively. Cells were cultivated in biological triplicates as described above. RNA isolation, Illumina sequencing and data processing was performed by the Göttingen Genomics Laboratory as described previously (Rosenbaum et al. 2022). For library preparation 1 μg DNA‐digested RNA was rRNA depleted and 40 ng were used. The total read counts (Fructose 1–3: 20,070,581, 19,357,916, 20,757,309; Fructose + Nitrate 1–3: 21,474,454, 18,734,385, 18,538,476) and the mapped read counts (Fructose 1–3: 11,815,815, 10,543,519, 12,033,574; Fructose + Nitrate 1–3: 15,299,748, 13,344,171, 13,373,173) resulted in mapping rates of 54.47%–58.87% (Fructose 1–3) and 71.23%–72.14% (Fructose + Nitrate 1–3), respectively. Genes with a log2‐fold change of +2/−2 and a *p*‐adjust value < 0.05 were considered differentially expressed. Raw reads have been deposited in the sequence read archive (SRA) under accession no. SRR34772502‐SRR34772507.

### 2.6 Preparation of Cell‐Free Extract, Cytoplasmic‐ and Membrane Fractions

All steps were performed under strictly anoxic conditions in an anoxic chamber (Coy Laboratory Products Inc., Grass Lake, Michigan, USA) filled with N_2_/H_2_ (96%–98%/2%–4% [v/v]). To prepare cell‐free extract, 500 mL cell culture was harvested in the late exponential growth phase by centrifugation for 15 min at 8500 rpm and 4°C (JA10/Avanti, Beckman Coulter GmbH, Krefeld, Germany). The supernatant was discarded and the cells were washed in lysis buffer (25 mM Tris–HCl, 20 mM MgSO_4_, 2 mM DTE, 4 μM resazurin, pH 7.5). The cells were centrifuged for 15 min at 8500 rpm and 4°C (JA25.50/Avanti, Beckman Coulter GmbH, Krefeld, Germany) and resuspended in 7 mL lysis buffer containing 0.4 mM phenylmethylsulphonyl fluoride (PMSF) and 0.1 mg mL^−1^ DNase I. Cell disruption was performed using a French Pressure Cell Press (SLM AMINCO; SLM Instruments Inc., Urbana, Illinois, USA) at a pressure of 110 MPa. Cell debris was sedimented by centrifugation for 25 min (Centrifuge 5417R; Eppendorf, Hamburg‐Eppendorf, Germany). The cell‐free extract was used for further experiments or separated into cytoplasmic and membrane fraction by centrifugation at 45,000 rpm for 45 min. For further use the membrane fraction was washed in lysis buffer. The cell‐free extract, cytoplasm and membrane fractions were stored at 4°C until use and the protein concentration was determined as described before (Bradford 1976).

### 2.7 Enzyme Assays

Enzyme activities were measured in 1.8 mL anoxic cuvettes (*d* = 0.5 cm) (Glasgerätebau Ochs, Germany) sealed with rubber stoppers, containing 1 mL assay buffer (50 mM Tris–HCl, 2 mM DTE, 4 μM Resazurin, pH 7.0) and a gas atmosphere of N_2_. The cuvettes were incubated at 37°C. Cell‐free extract, cytoplasm or membrane fractions were added to the cuvettes using a precision syringe (Pressure‐Lok; VICI precision sampling, Baton Rouge, Louisiana, USA), followed by reduced methyl viologen (MV) (10 mM) or anthraquinone‐2,6‐disulfonate (AQDS) (0.5 mM) as electron donor. The electron donor was reduced using sodium dithionite and the reaction was started by adding nitrate or nitrite (4 mM each) or hydroxylamine (5 mM) as substrate. To determine the enzyme activities, the oxidation of reduced MV or reduced AQDS was followed at 604 nm (*ε*MV = 13.9 mM^−1^ cm^−1^) or 408 nm (*ε*AQDS = 7.2 mM^−1^ cm^−1^), respectively.

### 2.8 Detection of Cytochromes

Cytochromes were detected after SDS‐PAGE by heme peroxidase activity staining. The samples must not have been boiled or treated with ß‐mercaptoethanol. For heme peroxidase staining, the gel was first dissolved in a freshly prepared solution of three parts 6.3 mM 3,3′,5,5′‐tetramethylbenzidine in methanol and 7 parts 0.25 M Na‐acetate buffer (pH 5.0) for 2 h under light protection. The reaction was started by the addition of 0.1% [v/v] 30% H_2_O_2_ and stopped by rinsing the gel in H_2_O. Heme‐containing proteins were stained turquoise blue.

### 2.9 Analytical Methods

Proteins were separated in 12% polyacrylamide gels via SDS‐PAGE and stained with Coomassie brilliant blue G250 (Schägger and von Jagow 1987).

### 2.10 Replicates, Statistical Analysis and Data Representation

All experiments were performed with at least two and in most cases three, independent biological replicates. Each biological replicate was initiated with a freshly prepared medium and inoculum. Each biological replicate included technical duplicates. As the results obtained from independent biological replicates showed only minor variation, data from one representative biological replicate are presented in the figures. Shown values represent means with standard deviation (SD).

## 3 Results

### 3.1 Nitrate Stimulates Growth and Decreases Acetate Production in the Acetogenic Bacterium *S. ovata* Under Heterotrophic and Autotrophic Conditions

**FIGURE 1:**
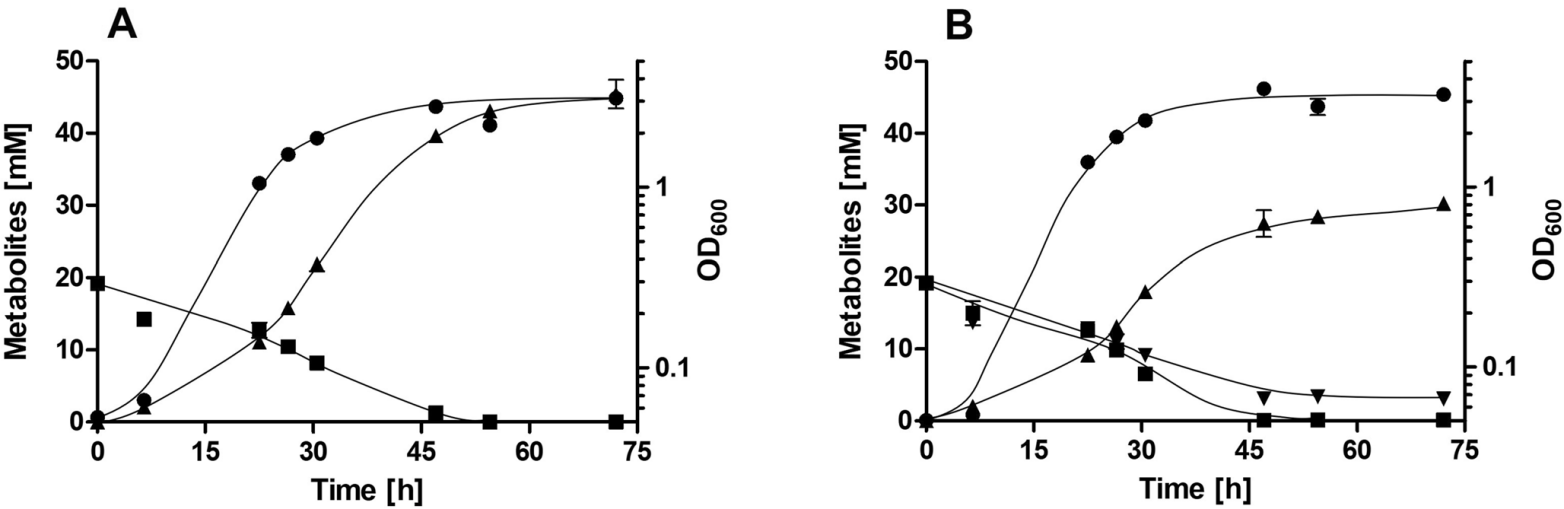
Growth of *Sporomusa ovata* on fructose in the presence of CO_2_ and metabolic profile in the presence or absence of nitrate. Cells were grown in bicarbonate‐buffered complex media with 20 mM fructose (A) and 20 mM fructose + 20 mM nitrate (B) under a N_2_/CO_2_ atmosphere (80:20 [v/v]). The optical density (●) was measured at 600 nm and fructose (■), nitrate (▼) and acetate (▲) concentrations were determined. Data represent one representative biological replicate (mean ± SD) (*n* = 2 independent experiments).

**FIGURE 2:**
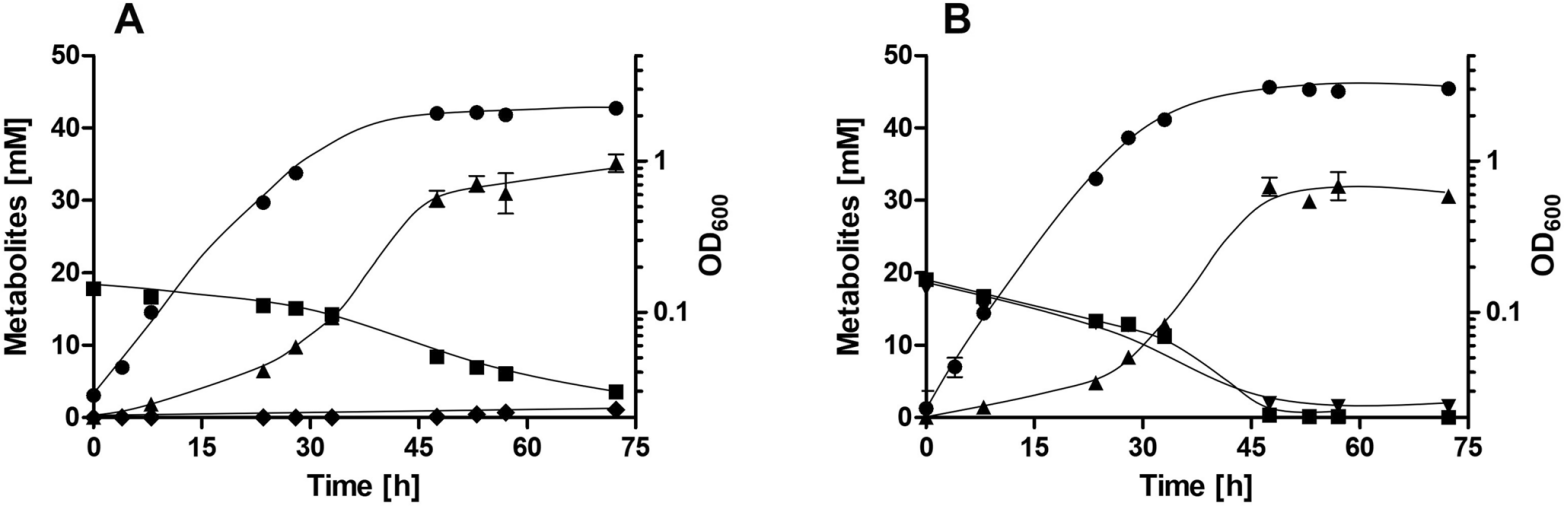
Growth of *Sporomusa ovata* on fructose and metabolic profile in the presence or absence of nitrate under CO_2_‐limited conditions. Cells were grown in bicarbonate‐free complex media with 100% N_2_ in the gas phase. The media was supplemented with 20 mM fructose (A) and 20 mM fructose + 20 mM nitrate (B). The optical density (●) was measured at 600 nm and fructose (■), nitrate (▼), acetate (▲) and ethanol (◆) concentrations were determined. Data represent one representative biological replicate (mean ± SD) (*n* = 2 independent experiments).

To study the effect of nitrate on growth of *S. ovata* , cells were grown on 20 mM fructose in bicarbonate‐buffered complex media in the absence or presence of 20 mM sodium nitrate. Cells grown in the absence of nitrate grew with a doubling time of 4 h, corresponding to a growth rate μ of 0.17 h^−1^ and reached a final OD_600_ of 3.1 (Figure 1A). In the presence of nitrate the final OD_600_ increased to 3.5 and the doubling time decreased to 3.7 h (*μ* = 0.19 h^−1^) (Figure 1B). Although the effect was modest, it clearly shows that nitrate stimulates heterotrophic growth. Furthermore, the metabolic profile of cells grown in the absence or presence of nitrate was analysed. Oxidation of one mole of fructose via glycolysis and pyruvate oxidation yields two moles of acetate; a third mole of acetate is produced in the WLP from two moles of CO_2_ and the four reducing equivalents gained from fructose oxidation. In the absence of nitrate, 45.4 mM acetate were produced from 19.2 mM fructose after 72 h (Figure 1A), giving an acetate/fructose ratio of 2.4. The presence of nitrate led to a decreased acetate concentration; only 30.2 mM acetate were produced from 19.1 mM fructose after 72 h, giving an acetate/fructose ratio of 1.6; at the same time 15.6 mM nitrate were consumed (Figure 1B). Nitrate reduction was also observed under CO_2_‐limited conditions. Cells grown in the absence of bicarbonate/CO_2_ grew slowly with a doubling time of 6.5 h (*μ* = 0.11 h^−1^) and the final OD_600_ was only 2.2 (Figure 2A). Acetate was the major product (35.1 mM); minor amounts of ethanol (1.1 mM) were produced after 72.3 h. Again, supplementation with nitrate led to an increased final OD_600_ of 3.1 and a decreased doubling time of 5.2 h (*μ* = 0.13 h^−1^) (Figure 2B). Also, the acetate concentration decreased to 30.6 mM after 72.3 h. The decrease in acetate/fructose ratio below 2 in both cases indicates that electrons coming from fructose oxidation were no longer funnelled into the WLP but towards nitrate; apparently, nitrate was the preferred electron acceptor under these conditions.

**FIGURE 3:**
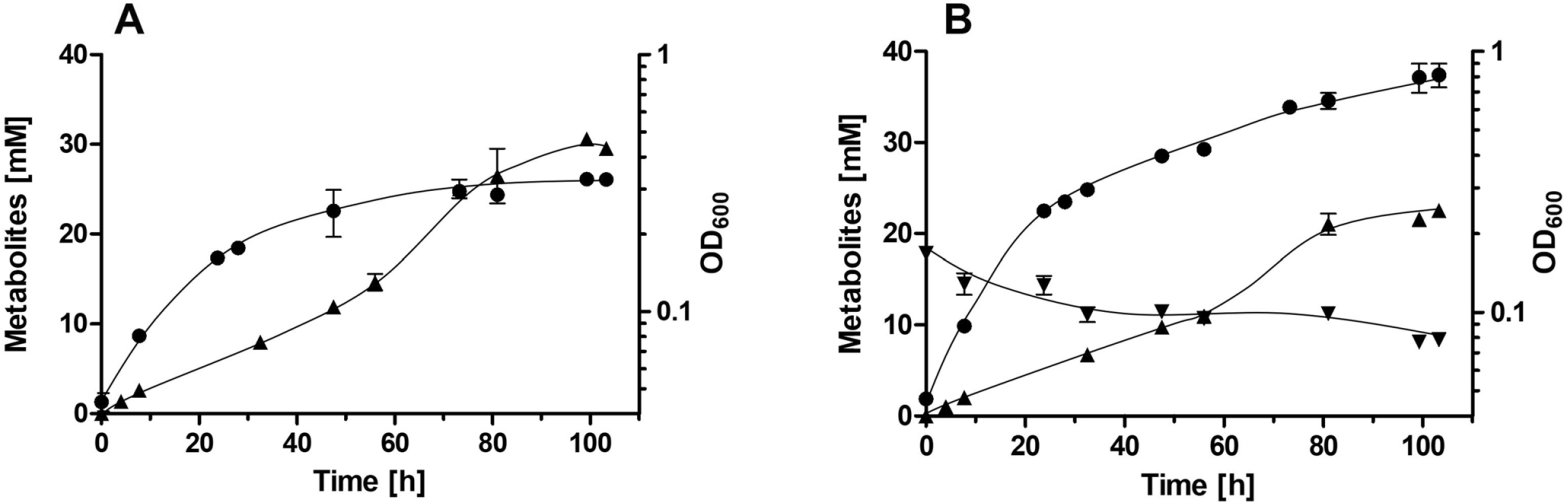
Growth of *Sporomusa ovata* on H_2_ + CO_2_ and metabolic profile in the presence or absence of nitrate. Cells were grown in bicarbonate‐buffered complex media with H_2_ + CO_2_ (80:20 [v/v]) (1 bar overpressure) (A) and H_2_ + CO_2_ + 20 mM nitrate (B). The optical density (●) was measured at 600 nm and nitrate (▼) and acetate (▲) concentrations were determined. Data represent one representative biological replicate (mean ± SD) (*n* = 2 independent experiments).

Next, we tested the effect of nitrate under autotrophic growth conditions. Under these conditions, acetate can only be formed from CO_2_. Cells were grown on H_2_ + CO_2_ (80:20 [v/v]) in the absence or presence of 20 mM sodium nitrate. Autotrophic conditions led to a final OD_600_ of 0.33, with a doubling time of 17.3 h (*μ* = 0.04 h^−1^) (Figure 3A). In the presence of nitrate, the doubling time decreased by 30% to 12.2 h (*μ* = 0.06 h^−1^) and the final OD_600_ increased by 246% to 0.81 (Figure 3B). In the absence of nitrate, 30.6 mM acetate was produced after 99.3 h (Figure 3A), whereas the addition of nitrate led to a production of only 24.5 mM acetate; about 50% of the nitrate was consumed (9.4 mM) (Figure 3B). Under autotrophic conditions, nitrate and CO_2_ were apparently used simultaneously as electron acceptors.

### 3.2 Metabolic Profile of Resting Cells of *S. ovata* in the Presence and Absence of Nitrate

**FIGURE 4:**
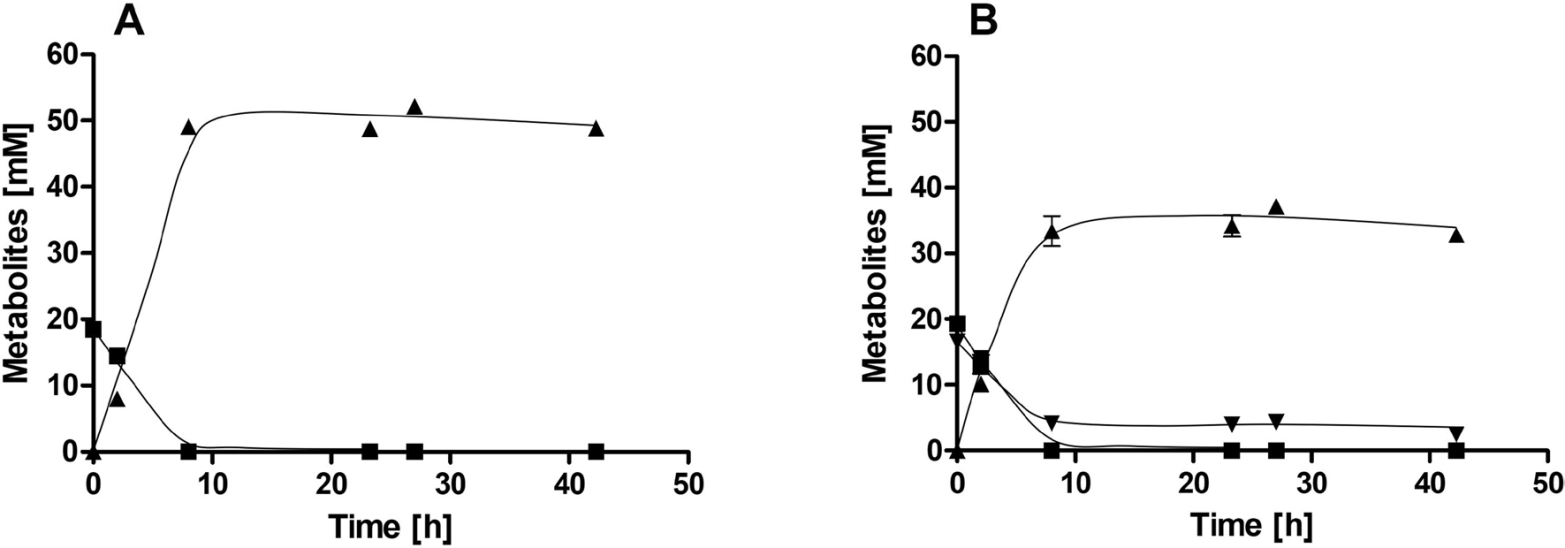
Fermentation of fructose in resting cells of *Sporomusa ovata* in the presence of CO_2_ and presence or absence of nitrate. Cells were grown in bicarbonate‐buffered complex medium with 20 mM fructose + 20 mM nitrate under a N_2_/CO_2_ atmosphere (80:20 [v/v]) and harvested in the early stationary growth phase. After washing, the cells were resuspended in 10 mL of cell suspension buffer (50 mM imidazole‐HCl, 20 mM MgSO_4_, 60 mM KHCO_3_, 2 mM DTE, 4.4 μM resazurin, pH 7) in 120 mL serum flasks under a N_2_/CO_2_ atmosphere at a total protein concentration of 1.5 mg mL^−1^. 20 mM fructose (A) or 20 mM fructose + 20 mM nitrate (B) was given to the cell suspensions. Fructose (■), acetate (▲) and nitrate (▼) were determined at each time point. Data represent one representative biological replicate (mean ± SD) (*n* = 2 independent experiments).

**FIGURE 5:**
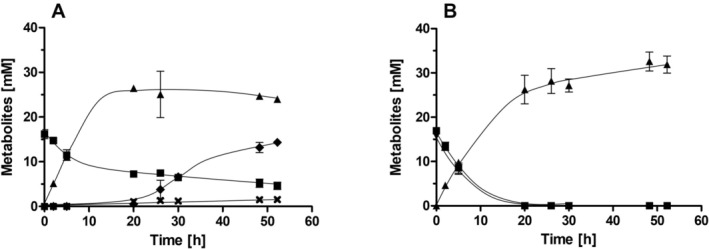
Fermentation of fructose in resting cells of *Sporomusa ovata* in the presence and absence of nitrate under CO_2_‐limited conditions. Cells were grown in CO_2_/bicarbonate‐free complex media with 20 mM fructose + 20 mM nitrate under a 100% N_2_ atmosphere and harvested in the early stationary growth phase. After washing, the cells were resuspended in 10 mL of cell suspension buffer (50 mM imidazole‐HCl, 20 mM MgSO_4_, 2 mM DTE, 4.4 μM resazurin, pH 7) in 120 mL serum flasks under a N_2_ atmosphere at a total protein concentration of 1.5 mg mL^−1^. 20 mM fructose (A) or 20 mM fructose + 20 mM nitrate (B) was given to the cell suspensions. Fructose (■), acetate (▲), nitrate (▼), lactate (◆) and ethanol (×) were determined at each time point. Data represent one representative biological replicate (mean ± SD) (*n* = 2 independent experiments).

To analyse the effect of nitrate on CO_2_ reduction, experiments with resting cells were performed. Resting cells do not convert CO_2_/carbon to biomass and thus, fermentation balances can be calculated more accurately. Resting cells of *S. ovata* performed homoacetogenesis from 20 mM fructose + CO_2_/bicarbonate. After 8 h, fructose was completely consumed, leading to a halt in acetate production. The ratio of fructose consumed and acetate produced was 1:2.8, with a carbon and electron balance of 94% (Figure 4A). When nitrate was present, again acetate was the only product from fructose (Figure 4B). The fructose:acetate ratio of resting cells with 20 mM fructose + CO_2_/bicarbonate + 20 mM nitrate was 1:1.9, indicating that the electrons from fructose oxidation via glycolysis were apparently used for the reduction of nitrate instead of CO_2_ via the WLP to generate a third mol of acetate (Figure 4B). Interestingly, in the absence of CO_2_/bicarbonate, fructose consumption was much slower and resting cells performed mixed acid fermentation and produced 1.5 mM ethanol and 14.4 mM lactate as side products (Figure 5A). Under CO_2_‐limited conditions the WLP can no longer work sufficiently, causing a redox imbalance because electrons from fructose oxidation can no longer be transferred to CO_2_. To “recycle” the available redox equivalents, the cells switch to mixed acid fermentation, producing ethanol and lactate as alternative electron sinks. After 42.3 h, 11.6 mM fructose were consumed and 24.0 mM acetate were produced as main product, leading to a fructose:acetate:lactate:ethanol ratio of 1:2.1:1.2:0.1 (Figure 5A). Again, acetate seems to solely stem from glycolysis and pyruvate oxidation. In the presence of nitrate, lactate and ethanol were no longer produced (Figure 5B). After 20 h, fructose and nitrate were depleted, leading to a halt in acetate production. 17.0 mM fructose and 15.7 mM nitrate were consumed and 31.9 mM acetate were produced, resulting in a fructose: acetate ratio of 1:1.9 (Figure 5B). Taken together, the experiments demonstrate that nitrate is preferred over CO_2_ as electron acceptor under heterotrophic conditions; the WLP is not engaged in the presence of nitrate.

### 3.3 H_2_ ‐Dependent Nitrate Reduction in *S. ovata*

**FIGURE 6:**
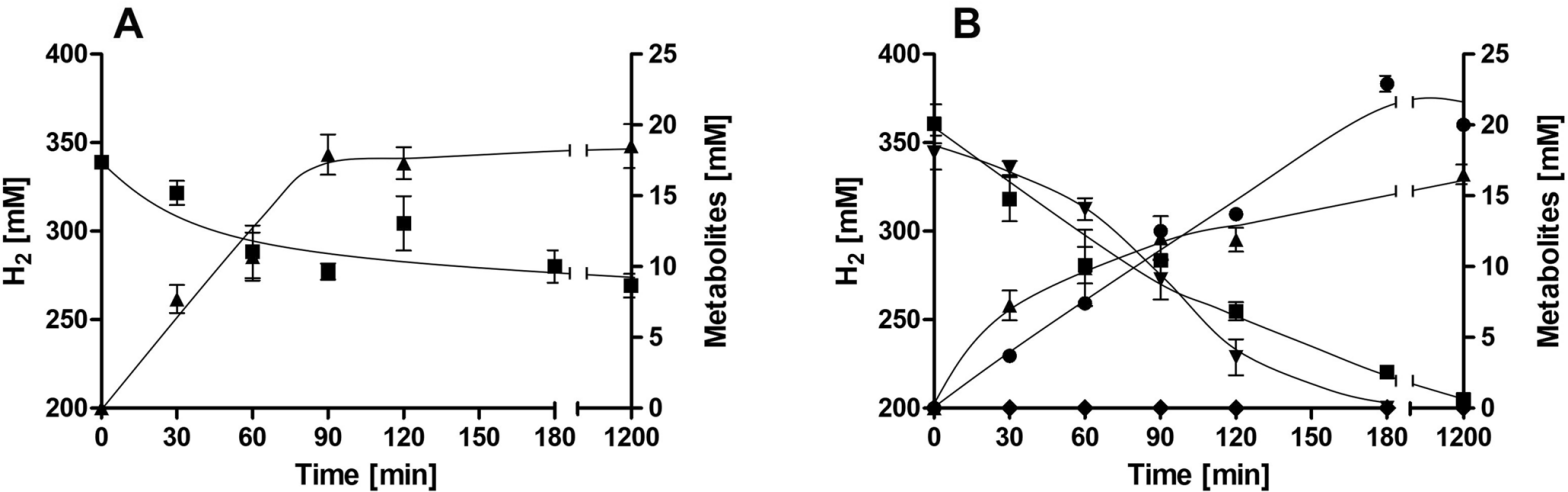
H_2_‐dependent CO_2_ and nitrate reduction by resting cells of *Sporomusa ovata* . Cells were grown in bicarbonate‐buffered complex medium media with 20 mM fructose + 20 mM nitrate under a N_2_/CO_2_ atmosphere (80:20 [v/v]) and harvested in the exponential growth phase. After washing, the cells were resuspended in 10 mL of cell suspension buffer (50 mM imidazole‐HCl, 20 mM MgSO_4_, 2 mM DTE, 4.4 μM resazurin, pH 7) in 120 mL serum flasks at a total protein concentration of 1.5 mg mL^−1^. In (A), the flasks contained a H_2_/CO_2_ (80:20 [v/v]) atmosphere. In (B), 20 mM nitrate was given to the cell suspensions. H_2_ (■), acetate (▲), nitrate (▼), ammonium (●) and nitrite (◆) were determined at each time point. Data represent one representative biological replicate (mean ± SD) (*n* = 3 independent experiments).

Next, nitrate respiration under autotrophic conditions was analysed in resting cells to gain insights into the electron flow during nitrate reduction. Under autotrophic conditions, resting cells of *S. ovata* produced acetate as the sole product from H_2_ + CO_2_ (Figure 6A). Within 20 h, 69.8 mM H_2_ were oxidised and 18.5 mM acetate was produced, resulting in a H_2_:acetate ratio of 3.8:1, which is consistent with the stoichiometry of autotrophic acetogenesis (Figure 6A). In the presence of nitrate, more hydrogen (155.7 mM) was oxidised after 20 h, but only 16.5 mM acetate was produced (Figure 6B). During the same time period, 18.1 mM nitrate was completely reduced to ammonium and nitrite accumulation was not detected (Figure 6B). Apparently, H_2_ serves as an electron donor for nitrate reduction to ammonium; in the presence of CO_2_ and nitrate, the available electrons are distributed between CO_2_ reduction and nitrate reduction at a ratio of 1:1.2. Under autotrophic conditions, CO_2_ and nitrate reduction occur simultaneously.

### 3.4 The *S. ovata* Genome Harbours a Nitrate Reduction Island

**FIGURE 7:**
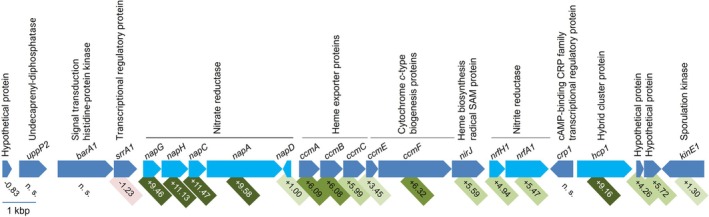
The nitrate reduction island in *Sporomusa ovata* and its upregulation by nitrate. Log2‐fold changes of transcriptome data from cells grown on fructose + nitrate compared to cells grown on fructose are indicated. n. s., not significant using a *p*‐adjust value of < 0.05 as threshold.

Inspection of the genome of *S. ovata* (accession number: CP146301; Böer et al. 2024) revealed a set of *nap* genes potentially coding for a periplasmic nitrate reductase complex, as well as additional genes related to cytochrome *c* and heme biosynthesis and genes responsible for periplasmic nitrite reduction (Figure 7). The *napGHCAD* cluster contains the 546 bp long *napG* gene (SOV_05710) coding for a 19.3 kDa protein. NapG is hydrophilic and has an N‐terminal signal sequence; therefore, NapG is most likely located in the periplasm. It contains three 4Fe‐4S clusters and is proposed to be part of the quinol dehydrogenase complex NapGH. Next to *napG*, the 798 bp *napH* (SOV_05720) encodes a 29.4 kDa protein containing four transmembrane helices. Therefore, NapH is most likely a membrane‐integral protein. It contains two 4Fe‐4S clusters and is proposed to be part of the quinol dehydrogenase complex NapGH. The *napC* gene (SOV_05730) consists of 537 bp and is proposed to encode a tetraheme cytochrome *c*‐containing quinol dehydrogenase. NapC has a predicted mass of 19.8 kDa and contains four heme‐binding motifs. It has one transmembrane helix and is, therefore, probably a membrane‐anchored protein. NapGH and NapC are involved in electron transfer from the quinol pool (Q‐pool) to the catalytic subunit (Brondijk et al. 2002; Kern and Simon 2008; Visser et al. 2016). NapGH shows amino acid sequence similarities up to 43% and NapC of 30% to *Escherichia coli* NapGH and NapC, respectively, both responsible for electron transfer from a quinol to the catalytic subunits of the nitrate reductase (Brondijk et al. 2004). The 2262 bp long *napA* gene (SOV_05740) is predicted to encode the catalytic subunit of the periplasmic nitrate reductase. NapA has an N‐terminal twin‐arginine translocation (TAT) signal sequence for protein translocation into the periplasm. It has a predicted mass of 83.6 kDa and contains a molybdopterin cofactor and is the site of nitrate reduction to nitrite. The last gene of the cluster is *napD* (SOV_05750), consisting of 537 bp and coding for an 8.8 kDa chaperone. NapD is likely responsible for the maturation of the catalytic subunit NapA. The *napGHCAD* gene cluster is followed by genes coding for heme exporter, as well as cytochrome *c* and heme biosynthesis proteins (SOV_05760‐05810) (Figure 7). Downstream are two genes coding for a putative periplasmic nitrite reductase NrfH1A1. The 474 bp *nrfH1* (SOV_05820) is predicted to code for a tetraheme cytochrome *c*, the small subunit of the nitrite reductase. NrfH1 has a predicted mass of 17.0 kDa and contains four heme‐binding motifs. It has one transmembrane helix and is, therefore, probably a membrane‐anchored protein. The 1290 bp *nrfA1* gene (SOV_05830) is predicted to encode a cytochrome *c*‐552 with a mass of 47.8 kDa. NrfA1 contains four heme‐binding motifs. It has a hydrophilic amino acid composition and an N‐terminal signal sequence and is, therefore, most likely a periplasmic enzyme. NrfA1 is proposed to form a stable, membrane‐associated complex with its electron donor NrfH1 to catalyse the six‐electron reduction of nitrite to ammonium (Einsle et al. 1999). Interestingly, an *hcp1* gene (1644 bp) (SOV_05850) is also localised downstream of these genes. The role of the hybrid cluster protein Hcp is discussed controversially (see discussion). It is often annotated as hydroxylamine reductase, but biochemical evidence also points to a role as NO reductase (Cole 2021; Hagen 2019, 2022; Wang et al. 2016). The GC content of the whole *S. ovata* genome is 43.5%, whereas the GC content of the nitrate reduction island (SOV_05710‐SOV_05850) is substantially higher at 53.7%. The higher GC content in the nitrate reduction island suggests that this region may have been acquired by horizontal gene transfer.

### 3.5 Genes Differentially Regulated by Nitrate

To analyse whether the expression of *nap* and possibly other genes is regulated by nitrate, the transcriptome of cells grown on fructose + nitrate was compared to cells grown on fructose in the absence of nitrate. Using a log2‐fold change (FC) of > + 2 and a *p*‐adjust value of < 0.05 as threshold, 1662 genes were differently regulated. Six hundred and seventy genes were upregulated and 992 genes were downregulated in the presence of nitrate. All the *nap* genes (SOV_05710–05750) were highly upregulated in nitrate‐grown cells (FC +9.46–+11.47) (chaperone *napD* FC +1.00) (Figure 7). Moreover, transcript levels of genes coding for the putative periplasmic nitrite reductase (SOV_05820–05830) (FC +4.94 to +5.47) and genes related to cytochrome *c* and heme biosynthesis (SOV_05760–05810) (FC +3.45 to +6.32) were also increased in the presence of nitrate (Figure 7). Furthermore, the transcript level of the *hcp1* gene (SOV_05850) was highly increased in nitrate‐grown cells (FC +9.16) (Figure 7). These data are in line with the hypothesis that nitrate is reduced to nitrite and further to ammonium. Transcript levels of different putative transporters were also increased in cells grown in the presence of nitrate. One of these upregulated transport systems is encoded by three genes (SOV_08090–08110) annotated as putative nitrate/nitrite ABC transport system, with log2‐fold changes between +2.09 and +3.35 and therefore a candidate for a nitrate transport system in *S. ovata* . It shows high amino acid sequence similarities up to 95.5% to other putative nitrate‐importing ABC transporters of the genus *Sporomusa* and 46% and 44% amino acid sequence similarity to annotated nitrate/sulfonate/bicarbonate ABC transporters of *Clostridium aceticum* and *Clostridium pasteurianum* , respectively.

Nitrate had no effect on the transcript levels of genes encoding the central metabolism of *S. ovata* . The genes coding for the formyl‐THF synthetase (*fhs*, SOV_14230), the methenyl‐THF cyclohydrolase (*fchA*, SOV_14190), the methylene‐THF dehydrogenase (*folD*, SOV_14200) and the methylene‐THF reductase (*metVF*, SOV_14350–14360) showed log2‐fold changes between −1.19 and +0.66. The genes encoding the key enzyme of the WLP, the CODH/ACS (*acsBA*, SOV_14240–14250), as well as the corrinoid/iron sulphur protein (*acsCD*, SOV_14260, SOV_14290), the corrinoid activation and regeneration protein (*acsV*, SOV_14270), the CoFeSP methyltransferase (*acsE*, SOV_14300), the CODH maturation factor (*cooC*, SOV_14220) and the CODH nickel‐insertion protein (*acsF*, SOV_14280) were also not differentially expressed in the presence of nitrate (FC −0.87 to +1.74). Furthermore, the transcript level of genes coding for the electron bifurcating hydrogenase (*hydCEDBA*, SOV_14560–14,600) (FC −0.65 to +1.14), the Rnf complex (*rnfCDGEAB*, SOV_14710–14760) (FC +0.33 to +1.16) and a F_O_F_1_‐ATP synthase (*atpIBEFHAGDC*, SOV_41450–41530) (FC −3.22 to +1.28) were not affected by the presence of nitrate. The same applies to the genes coding for the acetate kinase (*ack*, SOV_17600) (FC +0.31) and the phosphotransacetylase (*pta*, SOV_14090) (FC –0.13).

Among the most downregulated genes are several related to two‐component systems and quorum sensing (FC –6.45 to −2.08). Furthermore, the presence of flagella‐ and chemotaxis‐related genes among the most downregulated genes (FC –3.32 to −2.51) suggests reduced motility. Overall, these findings may indicate a potential stress response caused by the presence of nitrate. However, genes associated with the central carbon metabolism of *S. ovata* were not among the downregulated genes.

### 3.6 Membrane‐Bound Nitrate and Nitrite Reductase Activity in *S. ovata*

The experiments described so far suggest the presence of a nitrate and nitrite reductase in *S. ovata* . To search for these enzyme activities, cells were grown with 20 mM fructose in the presence of 20 mM sodium nitrate, harvested in the late‐exponential growth phase and cell‐free extract was prepared. Cytoplasm and membrane fractions were separated from each other. Reduced MV or reduced AQDS served as electron donors for the reductases. The electron donors were reduced with sodium dithionite and their substrate‐dependent oxidation was monitored at 604 or 408 nm, respectively. First, nitrate reductase activity was measured. The cell‐free extract had a specific MV‐dependent nitrate reductase activity of 1.9 ± 0.4 U mg^−1^. The specific nitrate reductase activity in the membrane fraction (5.3 ± 0.2 U mg^−1^) was 11‐fold higher compared to the cytoplasm (0.48 ± 0.01 U mg^−1^) (Table 1). The comparison of the total activities in cytoplasm and membrane fractions showed 1.8‐fold higher activity in membranes (44.5 U) than in the soluble fraction (24.9 U); 64% of the nitrate reductase activity was present in membranes (Table 1). Next, nitrite reductase activity was measured. With reduced MV as electron donor, the specific activity in the cell‐free extract was 6.1 ± 0.8 U mg^−1^. The membrane fraction showed 5.6‐fold higher specific nitrite reductase activity (3.4 ± 0.09 U mg^−1^) compared to the cytoplasm (0.6 ± 0.01 U mg^−1^) (Table 1). When total activities in the soluble and membrane fractions were compared, 73% of the nitrite reductase activity was found in the membranes (345.1 U), whereas 27% were found in the cytoplasm (128.1 U) (Table 1). Interestingly, with the reduced quinone analogue AQDS, nitrate and nitrite reductase activities were measurable only in the membrane fraction of nitrate‐grown cells; the specific nitrate reductase activity was 100 ± 20 mU mg^−1^ and nitrite reductase activity was 82.6 ± 2.4 mU mg^−1^ (Table 1). Nitrate and nitrite reductase activities were induced by the presence of nitrate and could not be observed in cells grown in the absence of nitrate. Furthermore, we observed hydroxylamine reductase activity exclusively in cells grown in the presence of nitrate. The cell‐free extract of these cells had a specific hydroxylamine reductase activity of 6.0 ± 0.1 U mg^−1^ with reduced MV as electron donor. Since it is known that nitrite reductases in bacteria like *E. coli* or *Wolinella succinogenes* (Clarke et al. 2004; Kern et al. 2011) can also reduce hydroxylamine, this enzyme activity is probably caused by the nitrite reductase from *S. ovata* .

**TABLE 1:** Nitrate and nitrite reductase activities of *S. ovata* cells grown in the presence of nitrate.

Enzyme activity^a^	Cell‐free extract	Cytoplasm	Membranes	
			Reduced methyl viologen^b^	Reduced AQDS^b^
Nitrate reductase	1.9 ± 0.4 U mg^−1^	0.48 ± 0.01 U mg^−1^ (24.9 U)	5.3 ± 0.2 U mg^−1^ (44.5 U)	100 ± 20 mU mg^−1^
Nitrite reductase	6.1 ± 0.8 U mg^−1^	0.6 ± 0.01 U mg^−1^ (128.1 U)	3.4 ± 0.09 U mg^−1^ (345.1 U)	82.6 ± 2.4 mU mg^−1^

Abbreviation: AQDS, anthraquinone‐2,6‐disulfonate.

^a^ All activities were determined as described in ‘Experimental Procedures’. Data shown as mean ± SD (*n* = 3 biological replicates). Each replicate included multiple measurements per assay.

^b^ Electron donor.

### 3.7 Nitrate Serves as Nitrogen Source in *S. ovata*

The above‐mentioned experiments are in line with the hypothesis that nitrate serves as an electron acceptor in *S. ovata* . In addition, nitrate could also serve as a nitrogen source. To test this hypothesis, cells were grown in minimal medium free of yeast extract and tryptone and without ammonium chloride; 20 mM fructose served as substrate. Several transfers in minimal media were performed to remove remaining nitrogen sources from the complex medium. In minimal media without ammonium, cells reached an OD_600_ of only 0.72. The addition of nitrate restored this growth deficit and led to growth comparable to that in minimal media with ammonium chloride; a final OD_600_ of 2.85 and 2.50 was reached, respectively (Figure S1). These data demonstrate that nitrate is also used as a nitrogen source by *S. ovata* . Inspection of the genome sequence of *S. ovata* revealed only the before‐mentioned gene cluster coding for a nitrate reductase (SOV_05710–05750) and no evidence for a second, assimilatory nitrate reductase was found.

### 3.8 Presence of Cytochromes in Nitrate‐Grown Cells

**FIGURE 8:**
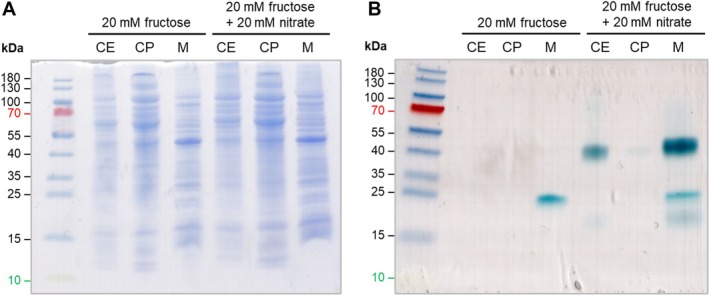
Presence of cytochromes in nitrate‐grown cells of *Sporomusa ovata* . SDS‐PAGE and in‐gel assay of heme peroxidase activity of cytochromes were prepared with cell‐free extract (CE), cytoplasm (CP) and membrane (M) fractions of cells grown on 20 mM fructose ± 20 mM nitrate. 25 μg CE, 50 μg CP and 50 μg M, respectively, were loaded on a 12% SDS‐PAGE gel and stained with Coomassie blue (A) or were incubated in a 6.3 mM 3,3′,5,5′‐tetramethylbenzidine‐containing solution (2 h under light protection) (B). The reaction was started by adding 0.1% [v/v] 30% H_2_O_2_. Turquoise‐stained proteins with heme peroxidase activity visible in the membrane fraction of cells grown in presence of nitrate correspond in mass to cytochrome *c*‐containing NapC (19.8 kDa) or a cytochrome *b*‐containing subunit of a hydrogenase and NrfH1A1 (17.0 kDa; 47.8 kDa) (B).

The nitrate as well as the nitrite reductase of *S. ovata* are predicted to contain cytochrome *c* as described for other nitrate and nitrite reductases (Kern and Simon 2009; Poock et al. 2002; Richardson et al. 2001). Therefore, we searched for cytochromes in *S. ovata* . Indeed, heme peroxidase staining of membranes from cells grown with fructose in the presence of nitrate revealed three proteins with heme peroxidase activity: a 45, a 25 and a 17 kDa protein (Figure 8B). The 45‐ and 17 kDa proteins could represent the cytochrome *c*‐containing subunits NrfA1 (47.8 kDa) and NrfH1 (17.0 kDa) of the periplasmic nitrite reductase. The 25 kDa protein could represent the cytochrome *c*‐containing subunit NapC of the nitrate reductase. In addition, a protein with a slightly smaller molecular mass was found in membranes of cells grown in the absence of nitrate; this protein could represent a component of the membrane‐bound, cytochrome *b*‐containing hydrogenase of *S. ovata* (Kremp et al. 2022).

## 4 Discussion

**FIGURE 9:**
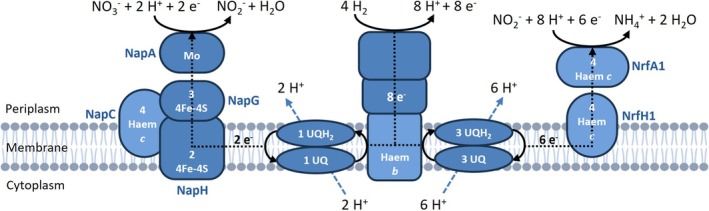
Proposed model of nitrate reduction to ammonium in *Sporomusa ovata* . Membrane‐bound, cytochrome *c*‐containing nitrate (Nap) and nitrite reductases (Nrf) of *S. ovata* receive the electrons for nitrate (NO_3_^−^) and nitrite reduction (NO_2_^−^), respectively, from the membrane‐embedded Q‐pool. The periplasmic cytochrome *b*‐containing [NiFe] hydrogenase generates scalar protons through hydrogen oxidation in the periplasm. The electrons are used to reduce coenzyme Q and, thereby, vectorial protons are translocated by the quinone cycle. A ΔpH and a Δψ are generated across the membrane. Fe‐S, iron–sulphur cluster; Mo, molybdopterin; NH_4_^+^, ammonium; UQ, ubiquinone.

Depending on the electron donor used, *S. ovata* utilised nitrate as a preferred electron acceptor or simultaneously with CO_2_, leading to changes in growth behaviour, product formation and electron distribution. Genome analysis revealed a nitrate reduction island encoding a periplasmic nitrate and nitrite reductase and transcriptome data showed strong nitrate‐dependent induction of expression of these genes as well as the neighbouring cytochrome *c* and heme biosynthesis genes. Enzyme activity assays supported these findings by demonstrating membrane‐associated nitrate and nitrite reductase activities that were detectable only in nitrate‐grown cells. In sum, our studies suggest a reduction of nitrate via nitrite to ammonium in *S. ovata* (Figure 9). The role of the Hcp is discussed controversially. Whereas some studies postulate a function as hydroxylamine reductase and hydroxylamine as intermediate in nitrate reduction (Hanson et al. 2013), Hcp is not a hydroxylamine reductase in *E. coli* . Instead, Hcp has been shown to detoxify NO, a compound produced from nitrite under high nitrate load (Figueiredo et al. 2013; Rowley et al. 2012; Wang et al. 2016). Although this is speculative for the moment, we also favour the latter for *S. ovata* . To achieve experimental insights into the function of Hcp in *S. ovata* , protein purification and activity assays would be particularly interesting for further studies.

Nitrate is long‐known as an electron acceptor for a membrane‐bound electron transport chain in facultative anaerobes such as *E. coli* as well as in strict anaerobes such as *Wolinella succinogenes* (Simon et al. 2000, 2003; Unden and Bongaerts 1997). In *E. coli* , two types of nitrate reductases are present—Nar and Nap. Both enzyme complexes are membrane‐bound. Nar is located on the cytoplasmic side of the inner membrane and Nap is located on the periplasmic side, as suggested here for *S. ovata* (Berks et al. 1995; Brondijk et al. 2002). Nar uses menaquinol as an electron donor and transfers electrons via cytochrome *b*. Since the electron transfer to nitrate is coupled with proton translocation across the membrane, Nar is involved in energy conservation through chemiosmotic ATP synthesis and serves as the primary respiratory nitrate reductase in *E. coli* (Richardson et al. 2001).

Nap receives electrons from menaquinol or ubiquinol and transfers them via cytochrome *c* to nitrate. Nitrate reduction in *E. coli* via Nap is not directly linked to proton translocation and thus fulfils a redox‐balancing role rather than an energy‐conservation role in the first place (Brondijk et al. 2004). Nap is not directly connected to energy conservation, but it indirectly assists in energy metabolism by receiving electrons for nitrate reduction from the reaction of a proton‐translocating enzyme complex like the NADH dehydrogenase in *E. coli* . NADH oxidation is coupled to H^+^ translocation across the membrane. This ion gradient is used to generate ATP and the electrons from NADH oxidation are shuttled through a Q‐pool to Nap for nitrate reduction (Brondijk et al. 2002, 2004; Potter et al. 1999).

In *W. succinogenes* , nitrate reduction is catalysed by the membrane‐bound periplasmic Nap‐type nitrate reductase, independent of a NapC subunit (Kern et al. 2007; Simon et al. 2003). Electrons are received from menaquinol and transferred through the menaquinol dehydrogenase complex NapGH (Kern et al. 2007; Kern and Simon 2008). The Nap system in *W. succinogenes* has not been demonstrated to be linked to direct proton translocation, but it is speculated to contribute indirectly to energy conservation via scalar proton consumption through menaquinol oxidation (Kern and Simon 2009).

Like in *E. coli* or *W. succinogenes* , the nitrate and nitrite reductases of *S. ovata* are membrane‐bound. Membrane‐bound reductase activities were observed with the quinone analogue AQDS, indicating that the membrane‐embedded Q‐pool is the electron donor for nitrate and nitrite reduction. A chemiosmotic potential (ion gradient) may be built up by scalar protons generated by the periplasmic hydrogenase of *S. ovata* and through consumption of protons for nitrate and nitrite reduction to ammonium (Figure 9). How energy is conserved by the membrane‐bound nitrate reduction pathway in *S. ovata* remains a challenging task for future studies.

In addition, non‐energy‐conserving, soluble nitrate reductases present in some anaerobes can increase cellular ATP yields indirectly (Emerson et al. 2019; Klask et al. 2022). During fermentation of carbohydrates, acetate formation from acetyl phosphate is the major ATP‐synthesising reaction. Under high electron pressure, acetyl‐CoA (the precursor of acetyl phosphate) serves as an electron acceptor with formation of acetaldehyde and ethanol and, thus, less ATP is formed via acetyl phosphate. If the electrons are redirected to, for example, nitrate, more acetate and thus more ATP is formed by substrate‐level phosphorylation (Thauer et al. 1977). In addition, there is a second, indirect effect. Like “caffeate respiration” in *A. woodii* , nitrate reduction in *C. ljungdahlii* is catalysed by soluble enzymes and driven by NADH oxidation. NAD^+^ is reduced by the electron‐bifurcating hydrogenase that reduces ferredoxin in equimolar amounts. Thus, for any NAD^+^ reduced, one ferredoxin is reduced and the reduced ferredoxin is reoxidized by the respiratory Rnf complex (Tremblay et al. 2012). Chemiosmotic ATP synthesis via Rnf and ATP synthase leads to the formation of 1.5 mol ATP, 138% more than in the absence of nitrate (Emerson et al. 2019).

Reduction of CO_2_ and nitrate in the presence of H_2_ occurred simultaneously in *C. ljungdahlii* (Emerson et al. 2019). The same was observed here with *S. ovata* . Transcript levels for nitrate and nitrite reductase were drastically increased in both *S. ovata* and *C. ljungdahlii* by the presence of nitrate. On the other hand, transcript levels for genes of the WLP were decreased 2‐ to 3.5‐fold in *C. ljungdahlii* , but this was not observed here in *S. ovata* . However, when the *C. ljungdahlii* culture was spiked with NO_3_^−^, transcript levels also remained unchanged (Emerson et al. 2019). Despite unchanged transcript levels of the WLP genes in *S. ovata* , the decreased acetate production indicates a redistribution of the electron flow. The WLP activity is most likely limited at the level of enzyme activity or even due to the availability of reducing equivalents. It is also possible, although not assumed for the nitrate reduction in *S. ovata* , that direct inhibition of the Ni‐dependent CODH/ACS complex by nitrate occurs, similar to what has been described in other non‐acetogenic bacteria (Katayama et al. 2025), thereby inhibiting acetogenesis via the WLP without any visible transcriptional changes. Nevertheless, it is plausible that there is a feedback between the WLP and nitrate reduction, allowing adaptation of the energy flow to changing electron acceptor availability.

It is obvious that the biochemistry, bioenergetics and physiological role of nitrate reduction to ammonium is different in the physiologically and phylogenetically quite different groups of acetogenic bacteria. This may stem from independent events leading to the uptake of different nitrate reduction genes from different donors to different acetogenic acceptors. Indeed, the nitrate reduction genes in *S. ovata* are clustered in one region, the nitrate reduction island, which has a different GC‐content than the genome. Transposons or insertion sequences flanking the island were not observed. These findings suggest that nitrate reduction in acetogenic bacteria may have arisen through multiple, independent horizontal gene transfer events rather than a single evolutionary origin.

## Author Contributions

**Lara M. Waschinger:** conceptualization, data curation, investigation, formal analysis, writing – original draft, writing – review and editing. **Anja Poehlein:** data curation, formal analysis, investigation. **Rolf Daniel:** data curation, formal analysis, investigation. **Florian P. Rosenbaum:** data curation, formal analysis. **Volker Müller:** conceptualization, data analyses, writing – original draft, writing – review and editing.

## Funding

This work was supported by the Deutsche Forschungsgemeinschaft via a Reinhart Koselleck Project.

## Conflicts of Interest

The authors declare no conflicts of interest.

## Supporting information


**Figure S1:** Nitrate can substitute ammonium as nitrogen source for *Sporomusa ovata*. Cells were grown in bicarbonate‐buffered ammonium‐free minimal media. After several transfers in minimal media the optical density of cells grown with 20 mM fructose (●) as substrate, in presence of nitrate (■) or ammonium chloride (▲) was measured at 600 nm. Data represent one representative biological replicate (mean ± SD) (*n* = 2 independent experiments).
**Table S1:** The most upregulated genes of *Sporomusa ovata* during growth in the presence of nitrate.
**Table S2:** The most downregulated protein‐coding genes of *Sporomusa ovata* during growth in the presence of nitrate.

## Data Availability

Transcriptome data have been deposited in the National Center for Biotechnology Information's (NCBI) Sequence Read Archive (SRA) under accession no. SRR34772502‐SRR34772507. All other data of this study are available from the corresponding author upon reasonable request.
