# Supplementary material for: A Cytochrome c‐Containing Periplasmic Nitrate Reductase in the Acetogen Sporomusa ovata
Source: Environ Microbiol. 2026 Jan 7;28(1):e70228. doi: 10.1111/1462-2920.70228 (PMC12780646; doi:10.1111/1462-2920.70228)
Supplement: Supplementary file 1 — Figure S1: Nitrate can substitute ammonium as nitrogen source for Sporomusa ovata. Cells were grown in bicarbonate‐buffered ammonium‐free minimal media. After several transfers in minimal media the optical density of cells grown with 20 mM fructose (●) as substrate, in presence of nitrate (■) or ammonium chloride (▲) was measured at 600 nm. Data represent one representative biological replicate (mean ± SD) (n = 2 independent experiments). Table S1: The most upregulated genes of Sporomusa ovata during growth in the presence of nitrate. Table S2: The most downregulated protein‐coding genes of Sporomusa ovata during growth in the presence of nitrate. [file EMI-28-e70228-s001.docx]

**Supplementary Data**

**A cytochrome *c*-containing periplasmic nitrate reductase**

**in the acetogen *Sporomusa ovata***

Lara M. Waschinger^1,3^, Anja Poehlein^2^, Rolf Daniel^2^, Florian P. Rosenbaum^1^ and Volker Müller^1#^

^1^*Molecular Microbiology & Bioenergetics, Institute of Molecular Biosciences, Johann Wolfgang Goethe University, Max-von-Laue Str. 9, D-60438 Frankfurt, Germany*

^2^*Georg August University Göttingen, Göttingen Genomics Laboratory, Institute of Microbiology and Genetics, D-37077 Göttingen, Germany*

*^3^ORCID iD: 0009-0003-8764-6946*

^#^*Corresponding author. Mailing address: Department of Molecular Microbiology & Bioenergetics, Institute of Molecular Biosciences, Johann Wolfgang Goethe University, Max-von-Laue Str. 9, D-60438 Frankfurt, Germany. Phone: 49-69-79829507. Fax: 49-69-79829306. E-mail:* [*vmueller@bio.uni-frankfurt.de*](mailto:vmueller@bio.uni-frankfurt.de)

**
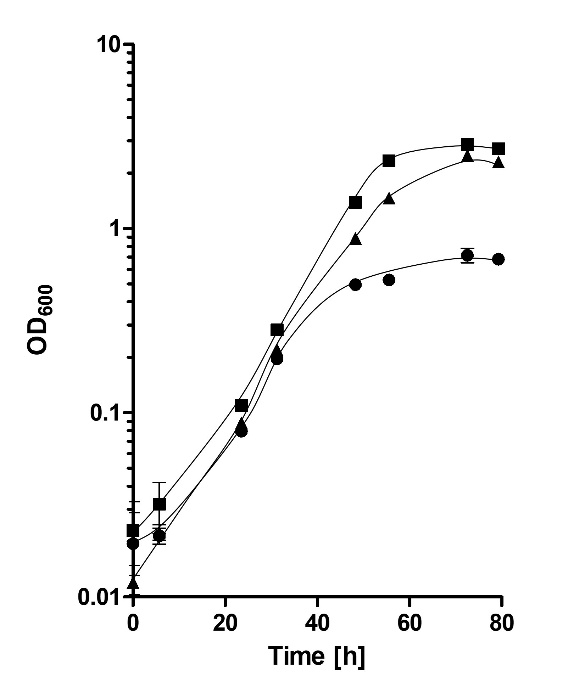
**

**Figure S1. Nitrate can substitute ammonium as nitrogen source for *S. ovata.*** Cells were grown in bicarbonate-buffered ammonium-free minimal media. After several transfers in minimal media the optical density of cells grown with 20 mM fructose (●) as substrate, in presence of nitrate (◼) or ammonium chloride (▲) was measured at 600 nm. Data represent one representative biological replicate (mean ± SD) (n = 2 independent experiments).

**Table S1. The most upregulated genes of *S. ovata* during growth in the presence of nitrate.**

| Gene | Annotation | Substrate | | Log2-fold change |
| --- | --- | --- | --- | --- |
|  |  | Fructose  + nitrate^1^ | Fructose^1^ |  |
| SOV_05730 | Cytochrome c-type protein NapC | 10028 | 2 | 11.47 |
| SOV_05720 | Putative electron transport protein NapH | 11702 | 2 | 11.13 |
| SOV_23490 | Transposase DDE domain protein | 168 | 0 | 10.77 |
| SOV_05740 | Periplasmic nitrate reductase precursor NapA | 30247 | 39 | 9.58 |
| SOV_05710 | quinol dehydrogenase periplasmic component NapG | 3596 | 5 | 9.46 |
| SOV_05850 | Hydroxylamine reductase Hcp1 | 19028 | 33 | 9.16 |
| SOV_44140 | Anaerobic dimethyl sulfoxide reductase chain B | 70 | 0 | 8.47 |
| SOV_07720 | Cupin domain protein | 275 | 1 | 8.36 |
| SOV_06060 | Anaerobic sulfite reductase subunit A | 19725 | 17 | 8.11 |
| SOV_28540 | Carboxymuconolactone decarboxylase family protein | 541 | 4 | 6.95 |
| SOV_38940 | transfer-messenger RNA, SsrA | 713217 | 6324 | 6.81 |
| SOV_40120 | ATP-dependent helicase HepA | 1970 | 19 | 6.73 |
| SOV_43890 | chromosome segregation protein | 1686 | 17 | 6.64 |
| SOV_05800 | Cytochrome c-type biogenesis protein CcmF1 | 33695 | 420 | 6.32 |
| SOV_05760 | Glutamine transport ATP-binding protein,  Heme transporter protein CcmA1 | 5362 | 78 | 6.09 |
| SOV_05770 | Heme transporter protein CcmB1 | 20531 | 110 | 6.08 |
| SOV_47320 | Cornifin (SPRR) family protein | 27 | 0 | 6.07 |
| SOV_05780 | Heme transporter protein CcmC1 | 7216 | 112 | 5.99 |
| SOV_38480 | Chloroplast import component protein (Tic20) | 15894 | 17 | 5.88 |
| SOV_22320 | Ribosomal large subunit pseudouridine synthase D | 1163 | 20 | 5.87 |
| SOV_00440 | N-acetylmuramoyl-L-alanine amidase LytC precursor | 423 | 7 | 5.82 |
| SOV_08480 | Cystathionine gamma-lyase | 232 | 4 | 5.79 |
| SOV_24640 | Carbon starvation protein A | 16957 | 311 | 5.77 |
| SOV_36970 | Bacterial regulatory protein, tetR family | 224 | 5 | 5.72 |
| SOV_06070 | Anaerobic sulfite reductase subunit B | 9979 | 50 | 5.68 |
| SOV_01740 | EamA-like transporter family protein | 26 | 0 | 5.67 |
| SOV_17120 | Helix-turn-helix domain protein | 10 | 0 | 5.64 |
| SOV_05810 | Antilisterial bacteriocin subtilosin biosynthesis protein AlbA, Heme synthase | 31621 | 289 | 5.59 |
| SOV_32520 | Cystathionine beta-lyase PatB | 3334 | 71 | 5.56 |
| SOV_06080 | Anaerobic sulfite reductase subunit C | 4618 | 92 | 5.54 |
| SOV_46140 | Energy-coupling factor transporter transmembrane protein EcfT | 12778 | 70 | 5.48 |
| SOV_05830 | Cytochrome c-552 precursor, NrfA1 | 101473 | 2213 | 5.47 |
| SOV_28180 | Heme/hemopexin transporter protein HuxB precursor | 429 | 10 | 5.43 |
| SOV_41720 | Cyclic di-GMP phosphodiesterase response regulator RpfG | 1448 | 34 | 5.41 |
| SOV_13740 | Ferrous-iron efflux pump FieF | 470 | 11 | 5.38 |
| SOV_17030 | HTH-type transcriptional regulator PuuR | 786 | 20 | 5.34 |
| SOV_27490 | lineage-specific thermal regulator protein | 1066 | 27 | 5.29 |
| SOV_46150 | Energy-coupling factor transporter ATP-binding protein EcfA3 | 3377 | 87 | 5.26 |
| SOV_31250 | putative HTH-type transcriptional regulator YttP | 1018 | 27 | 5.23 |
| SOV_05320 | HTH-type transcriptional regulator CynR | 515 | 13 | 5.22 |
| SOV_33720 | Macrolide export protein MacA | 20 | 0 | 5.15 |
| SOV_02780 | UDP-N-acetylmuramoyl-L-alanyl-D-glutamate-L-lysine ligase | 129 | 3 | 5.15 |
| SOV_06950 | Na(+)/H(+) antiporter NhaD | 2064 | 58 | 5.15 |
| SOV_38590 | Exoglucanase B precursor | 1825 | 51 | 5.15 |
| SOV_08130 | Na(+)-translocating NADH-quinone reductase subunit F | 55 | 2 | 5.08 |
| SOV_04140 | 50S ribosomal protein L24 | 45506 | 6388 | 5.02 |
| SOV_29690 | Glutathione import ATP-binding protein GsiA | 2418 | 73 | 5.01 |
| SOV_05580 | Biopolymer transport protein ExbB | 116 | 3 | 4.94 |
| SOV_05820 | Cytochrome c-type protein NrfH1 | 25882 | 839 | 4.94 |
| SOV_01240 | 2-aminoadipate transaminase | 2583 | 84 | 4.93 |
| SOV_12550 | Ribosomal large subunit pseudouridine synthase D | 675 | 22 | 4.89 |
| SOV_00420 | D-alanyl-D-alanine carboxypeptidase precursor | 734 | 25 | 4.86 |
| SOV_49560 | Tetraprenyl-beta-curcumene synthase | 1215 | 42 | 4.86 |
| SOV_03170 | CHAD domain protein | 4388 | 152 | 4.85 |
| SOV_02690 | putative 4-amino-4-deoxy-L-arabinose-phosphoundecaprenol flippase subunit ArnF | 850 | 31 | 4.77 |
| SOV_29050 | Putative DNA ligase-like protein | 60 | 2 | 4.74 |
| SOV_08470 | Cystathionine beta-lyase PatB | 369 | 13 | 4.74 |
| SOV_53110 | Vitamin B12 dependent methionine synthase, activation domain | 16199 | 55 | 4.70 |
| SOV_30650 | Lipase (class 3) | 3696 | 143 | 4.69 |
| SOV_30630 | Efflux pump membrane transporter BepE | 3122 | 120 | 4.69 |
| SOV_29020 | N-acetylmuramoyl-L-alanine amidase LytC precursor | 6 | 0 | 4.63 |
| SOV_43440 | Colicin I receptor precursor | 311 | 12 | 4.62 |
| SOV_53120 | Methionine synthase | 7625 | 312 | 4.61 |
| SOV_48050 | Ammonium transporter NrgA4 | 546 | 22 | 4.58 |
| SOV_04080 | 50S ribosomal protein L22 | 29471 | 16109 | 4.49 |
| SOV_09420 | GTP pyrophosphokinase YjbM | 465 | 20 | 4.48 |
| SOV_28120 | Heme/hemopexin transporter protein HuxB precursor | 31 | 1 | 4.48 |
| SOV_36800 | D-methionine-binding lipoprotein MetQ precursor | 2154 | 97 | 4.47 |
| SOV_04320 | 50S ribosomal protein L17 | 64350 | 3056 | 4.39 |
| SOV_45530 | Adenylosuccinate lyase | 18687 | 243 | 4.39 |
| SOV_42750 | Glycolate permease GlcA | 143 | 7 | 4.33 |
| SOV_10820 | 2-oxoglutarate carboxylase large subunit | 19455 | 317 | 4.33 |
| SOV_30540 | N-acetylmuramoyl-L-alanine amidase LytC precursor | 12800 | 505 | 4.32 |
| SOV_44690 | Sodium/glucose cotransporter | 695 | 36 | 4.25 |
| SOV_00090 | Putative L,D-transpeptidase YkuD | 8 | 0 | 4.25 |
| SOV_12660 | HutD | 729 | 38 | 4.23 |
| SOV_37530 | Glycolate permease GlcA | 645 | 35 | 4.17 |
| SOV_15660 | HTH-type transcriptional repressor AseR | 1454 | 81 | 4.15 |
| SOV_44600 | ASCH domain protein | 9409 | 296 | 4.14 |
| SOV_48890 | Transposase DDE domain protein | 1000 | 47 | 4.14 |
| SOV_45840 | 30S ribosomal protein S1 | 6147 | 352 | 4.12 |
| SOV_29490 | Heme/hemopexin-binding protein precursor | 16146 | 71 | 4.11 |
| SOV_29530 | putative protein kinase UbiB | 11 | 0 | 4.09 |
| SOV_27300 | Chaperone protein HtpG | 32954 | 1942 | 4.08 |
| SOV_19870 | HIT-like protein | 28665 | 1200 | 4.08 |
| SOV_33690 | 4'-phosphopantetheinyl transferase sfp | 150 | 8 | 4.07 |
| SOV_23210 | Methylmalonyl-CoA carboxyltransferase 5S subunit | 16777 | 581 | 4.06 |
| SOV_30440 | Phytochrome-like protein cph2 | 3596 | 220 | 4.02 |
| SOV_27970 | Bicarbonate transport ATP-binding protein CmpD | 17 | 1 | 3.99 |
| SOV_00590 | Alpha/beta hydrolase family protein | 112 | 7 | 3.98 |
| SOV_29660 | Periplasmic dipeptide transport protein precursor | 2015 | 126 | 3.95 |
| SOV_07820 | Chaperone protein dnaK2 | 100505 | 6537 | 3.94 |
| SOV_23920 | putative protease YhbU precursor | 6457 | 425 | 3.92 |
| SOV_24050 | Transposase DDE domain protein | 5550 | 366 | 3.91 |
| SOV_33260 | Calcineurin-like phosphoesterase superfamily domain protein | 10 | 0 | 3.87 |
| SOV_31100 | Branched-chain amino acid transport system / permease component | 489 | 33 | 3.86 |
| SOV_23220 | Iron hydrogenase 1 | 71 | 5 | 3.85 |
| SOV_44870 | Sporulation kinase E | 736 | 51 | 3.85 |
| SOV_29500 | Colicin I receptor precursor | 395 | 28 | 3.84 |
| SOV_29700 | putative permease | 186 | 12 | 3.83 |
| SOV_45000 | Lichenan-specific phosphotransferase enzyme IIA component | 11061 | 130 | 3.79 |
| SOV_36140 | lipoprotein NlpI | 14 | 1 | 3.79 |
| SOV_11750 | Cytosine deaminase | 2631 | 108 | 3.78 |
| SOV_29720 | 3-alpha-(or 20-beta)-hydroxysteroid dehydrogenase | 366 | 27 | 3.77 |
| SOV_29130 | Cyclic di-GMP phosphodiesterase response regulator RpfG | 2413 | 177 | 3.77 |
| SOV_33700 | putative efflux pump membrane transporter TtgB | 28 | 2 | 3.76 |
| SOV_30610 | Fatty acid metabolism regulator protein | 1224 | 90 | 3.74 |
| SOV_36440 | Anti-sigma-B factor antagonist | 57 | 4 | 3.74 |
| SOV_30220 | Nickel-binding periplasmic protein precursor | 206 | 15 | 3.74 |
| SOV_37100 | Outer membrane efflux protein | 41 | 3 | 3.73 |
| SOV_37430 | Acyl-coenzyme A thioesterase PaaI | 148 | 11 | 3.73 |
| SOV_25070 | Periplasmic [NiFeSe] hydrogenase large subunit | 43 | 3 | 3.71 |
| SOV_31570 | Cysteine-rich secretory protein family protein | 449 | 35 | 3.69 |
| SOV_48510 | PhoH-like protein | 47 | 3 | 3.67 |
| SOV_03180 | Processive diacylglycerol beta-glucosyltransferase | 1833 | 144 | 3.65 |
| SOV_28610 | Phosphomethylpyrimidine synthase | 343 | 27 | 3.64 |
| SOV_28110 | Heme/hemopexin-binding protein precursor | 123 | 9 | 3.64 |
| SOV_29480 | Heme/hemopexin transporter protein HuxB precursor | 325 | 26 | 3.64 |
| SOV_31000 | Zinc dependent phospholipase C | 160 | 13 | 3.62 |
| SOV_29030 | Oligoendopeptidase F, plasmid | 729 | 59 | 3.62 |
| SOV_45410 | General stress protein 39 | 144 | 11 | 3.60 |
| SOV_45550 | putative HTH-type transcriptional regulator YybR | 96 | 8 | 3.60 |
| SOV_25640 | colicin uptake protein TolQ | 162 | 13 | 3.60 |
| SOV_37920 | Spore germination protein A1 | 25 | 2 | 3.59 |
| SOV_09970 | mRNA interferase EndoA | 5856 | 485 | 3.59 |
| SOV_43410 | Sensor protein SrrB | 1045 | 87 | 3.58 |
| SOV_31270 | Multidrug export protein EmrB | 1662 | 138 | 3.57 |
| SOV_46250 | Methyltransferase domain protein | 25 | 2 | 3.55 |
| SOV_15150 | Putative multidrug export ATP-binding/permease protein | 11 | 1 | 3.55 |
| SOV_30450 | 2,3-diketo-L-gulonate-binding periplasmic protein YiaO precursor | 652 | 56 | 3.52 |
| SOV_51700 | Calcium-transporting ATPase 1 | 1161 | 100 | 3.52 |
| SOV_41880 | Sodium/proline symporter | 20454 | 673 | 3.51 |
| SOV_14460 | Formate dehydrogenase H | 76183 | 12643 | 3.50 |
| SOV_28480 | putative FAD-linked oxidoreductase | 257 | 23 | 3.49 |
| SOV_21360 | putative metallo-hydrolase | 2131 | 189 | 3.49 |
| SOV_32870 | Quinoprotein glucose dehydrogenase B precursor | 82 | 7 | 3.49 |
| SOV_15410 | 3',5'-cyclic adenosine monophosphate phosphodiesterase CpdA | 1125 | 101 | 3.47 |
| SOV_37760 | transcriptional regulator BetI | 491 | 45 | 3.47 |
| SOV_05790 | Cytochrome c-type biogenesis protein CcmE1 | 1006 | 92 | 3.45 |
| SOV_02680 | Acyltransferase family protein | 249 | 22 | 3.45 |
| SOV_17850 | 50S ribosomal protein L19 | 25719 | 2343 | 3.44 |
| SOV_07040 | dihydrolipoamide dehydrogenase | 7850 | 312 | 3.43 |
| SOV_37060 | Efflux pump membrane transporter BepE | 377 | 35 | 3.42 |
| SOV_32010 | Putative peroxiredoxin | 41 | 3 | 3.42 |
| SOV_03110 | L-lysine 2,3-aminomutase | 20008 | 1870 | 3.41 |
| SOV_06810 | Flagellar hook-associated protein 1 | 8 | 0 | 3.40 |
| SOV_28550 | Dihydrolipoyllysine-residue acetyltransferase component of pyruvate dehydrogenase complex | 989 | 94 | 3.38 |
| SOV_29680 | Oligopeptide transport system permease protein OppC | 750 | 70 | 3.36 |
| SOV_20580 | N-acyltransferase YncA | 156 | 15 | 3.36 |
| SOV_13800 | Iron-sulfur cluster carrier protein | 14724 | 579 | 3.35 |
| SOV_15920 | Heme/hemopexin transporter protein HuxB precursor | 63 | 6 | 3.35 |
| SOV_08110 | taurine transporter substrate binding subunit,  putative nitrate/nitrite transport system | 40 | 4 | 3.35 |
| SOV_49550 | Exopolyphosphatase | 2314 | 226 | 3.35 |
| SOV_30560 | Carbon monoxide dehydrogenase 2 | 307 | 30 | 3.34 |
| SOV_09710 | Group II intron-encoded protein LtrA | 474 | 46 | 3.33 |
| SOV_12570 | Glycolate permease GlcA | 840 | 83 | 3.33 |
| SOV_31110 | Branched-chain amino acid transport system / permease component | 470 | 47 | 3.31 |
| SOV_36460 | bacteriophage N4 adsorption protein B | 2648 | 267 | 3.30 |
| SOV_37000 | Multidrug resistance protein MdtC | 226 | 23 | 3.30 |
| SOV_44940 | Homoserine/homoserine lactone efflux protein | 944 | 95 | 3.30 |
| SOV_29240 | Macrolide export protein MacA | 547 | 55 | 3.29 |
| SOV_30640 | LPS-assembly protein LptD | 679 | 70 | 3.26 |
| SOV_39230 | Membrane protein of unknown function | 3496 | 364 | 3.26 |
| SOV_07100 | Long-chain-fatty-acid-CoA ligase | 17755 | 1846 | 3.26 |
| SOV_45880 | Cell division protein FtsA | 4455 | 466 | 3.25 |
| SOV_27470 | Glyoxalase-like domain protein | 24 | 2 | 3.24 |
| SOV_31090 | Ribose import ATP-binding protein RbsA | 1457 | 154 | 3.23 |
| SOV_11600 | site-specific tyrosine recombinase XerD | 8 | 0 | 3.21 |
| SOV_44890 | B12 binding domain protein | 130 | 13 | 3.21 |
| SOV_38440 | Phytochrome-like protein cph1 | 122 | 13 | 3.20 |
| SOV_00010 | Chromosomal replication initiator protein DnaA | 2714 | 295 | 3.20 |
| SOV_16440 | Cupin domain protein | 15626 | 128 | 3.19 |
| SOV_28170 | Heme/hemopexin-binding protein precursor | 2971 | 323 | 3.19 |
| SOV_06900 | Sensor protein SrrB | 9830 | 504 | 3.19 |
| SOV_06030 | Homoaconitase large subunit | 5023 | 548 | 3.19 |
| SOV_07090 | FmdE, Molybdenum formylmethanofuran dehydrogenase operon | 7113 | 783 | 3.18 |
| SOV_01570 | Ammonium transporter NrgA2 | 204 | 22 | 3.16 |
| SOV_06930 | Aspartate aminotransferase | 3286 | 365 | 3.15 |
| SOV_03130 | Alkaline phosphatase synthesis sensor protein PhoR | 457 | 51 | 3.14 |
| SOV_42290 | Putative N-acetyl-LL-diaminopimelate aminotransferase | 1649 | 187 | 3.13 |
| SOV_14540 | DRTGG domain protein | 12567 | 1447 | 3.11 |
| SOV_20050 | Regulatory protein BlaR1 | 304 | 34 | 3.11 |
| SOV_05190 | Glutamine-fructose-6-phosphate aminotransferase [isomerizing] | 7853 | 908 | 3.11 |
| SOV_26900 | Primosomal protein N' | 5910 | 679 | 3.11 |
| SOV_09380 | putative membrane protein | 303 | 34 | 3.10 |
| SOV_33640 | Lysozyme M1 precursor | 52 | 6 | 3.10 |
| SOV_37070 | Putative universal stress protein | 32 | 3 | 3.09 |
| SOV_17500 | Group II intron-encoded protein LtrA | 1472 | 171 | 3.09 |
| SOV_30770 | Nickel and cobalt resistance protein CnrA | 34 | 4 | 3.09 |
| SOV_08460 | Putative aminotransferase | 8956 | 15984 | 3.08 |
| SOV_21860 | putative inorganic polyphosphate/ATP-NAD kinase | 2127 | 249 | 3.08 |
| SOV_38290 | Limonene hydroxylase | 6921 | 173 | 3.06 |
| SOV_49190 | Peptidase C39 family protein | 64 | 7 | 3.05 |
| SOV_06940 | Glucosyl-3-phosphoglycerate synthase | 15792 | 373 | 3.05 |
| SOV_45500 | Response regulator rcp1 | 128 | 15 | 3.04 |
| SOV_19820 | Chaperone protein DnaK | 39689 | 4806 | 3.04 |
| SOV_28060 | Bifunctional hemolysin/adenylate cyclase precursor | 18 | 2 | 3.02 |
| SOV_03740 | RNA polymerase sigma-H factor | 13939 | 6586 | 3.02 |
| SOV_35390 | Inner membrane transport permease YbhS | 379 | 46 | 3.02 |
| SOV_44150 | Acetylene hydratase | 545 | 67 | 3.02 |
| SOV_18360 | Soluble lytic murein transglycosylase precursor | 4390 | 547 | 3.00 |
| SOV_28510 | Octanoyltransferase | 281 | 35 | 3.00 |
| SOV_30760 | Efflux pump membrane transporter BepE | 196 | 24 | 3.00 |
| SOV_28500 | Lipoate-protein ligase LplJ | 304 | 39 | 2.95 |
| SOV_46030 | Methionine aminotransferase | 10622 | 356 | 2.94 |
| SOV_53030 | Murein hydrolase activator EnvC precursor | 7149 | 931 | 2.94 |
| SOV_30990 | Processive diacylglycerol beta-glucosyltransferase | 306 | 40 | 2.91 |
| SOV_45590 | HTH-type transcriptional regulator ImmR | 118 | 15 | 2.90 |
| SOV_31260 | putative multidrug resistance protein EmrK | 939 | 123 | 2.89 |
| SOV_28530 | Lipoyl synthase | 392 | 52 | 2.89 |
| SOV_17570 | Sporulation integral membrane protein YlbJ | 309 | 41 | 2.88 |
| SOV_48710 | Gamma-DL-glutamyl hydrolase precursor | 121 | 15 | 2.88 |
| SOV_07540 | Tyrosine recombinase XerC | 386 | 51 | 2.87 |
| SOV_06630 | Transposase DDE domain protein | 407 | 53 | 2.87 |
| SOV_33910 | Putative multidrug export ATP-binding/permease protein | 15 | 2 | 2.87 |
| SOV_27960 | Putative aliphatic sulfonates transport permease protein SsuC | 12 | 1 | 2.86 |
| SOV_10830 | 2-oxoglutarate carboxylase small subunit | 4245 | 582 | 2.86 |
| SOV_30620 | Macrolide export protein MacA | 411 | 55 | 2.86 |
| SOV_18030 | Vitamin B12 transporter BtuB precursor | 30070 | 296 | 2.86 |
| SOV_30160 | FmdE, Molybdenum formylmethanofuran dehydrogenase operon | 15154 | 92 | 2.85 |
| SOV_42760 | Rubrerythrin-1 | 9161 | 1267 | 2.84 |
| SOV_38640 | Energy-coupling factor transporter transmembrane protein EcfT | 69 | 9 | 2.84 |
| SOV_23910 | Acetylornithine aminotransferase | 18436 | 598 | 2.82 |
| SOV_29060 | putative DNA repair protein YkoV | 19 | 2 | 2.82 |
| SOV_46470 | Betaine reductase complex component B subunit beta | 727 | 100 | 2.82 |
| SOV_28470 | Anaerobic glycerol-3-phosphate dehydrogenase subunit C | 148 | 20 | 2.79 |
| SOV_49880 | Transposase DDE domain protein | 2042 | 294 | 2.79 |
| SOV_21850 | 16S/23S rRNA (cytidine-2'-O)-methyltransferase TlyA | 1749 | 252 | 2.79 |
| SOV_41990 | Carbapenem-hydrolyzing beta-lactamase BlaB-1 precursor | 265 | 38 | 2.76 |
| SOV_05420 | Integrase core domain protein | 5825 | 858 | 2.76 |
| SOV_19080 | putative HTH-type transcriptional regulator YtcD | 44 | 6 | 2.76 |
| SOV_43430 | Multifunctional cyclase-dehydratase-3-O-methyl transferase TcmN | 136 | 19 | 2.75 |
| SOV_41080 | Putative multidrug export ATP-binding/permease protein | 7792 | 14770 | 2.75 |
| SOV_24020 | putative amino-acid metabolite efflux pump | 615 | 90 | 2.75 |
| SOV_07530 | Tyrosine recombinase XerC | 351 | 51 | 2.74 |
| SOV_06040 | Citrate synthase 2 | 8590 | 256 | 2.72 |
| SOV_52780 | MazG-like family protein | 742 | 112 | 2.71 |
| SOV_31640 | Transcriptional regulator YqjI | 340 | 51 | 2.71 |
| SOV_25520 | Multidrug-efflux transporter 1 regulator | 109 | 16 | 2.69 |
| SOV_09570 | Dimethyl sulfoxide reductase DmsA precursor | 327 | 50 | 2.68 |
| SOV_50720 | Holin family protein | 15 | 2 | 2.68 |
| SOV_39910 | Flagellar hook-associated protein 1 | 9355 | 1147 | 2.67 |
| SOV_36430 | Putative methyl-accepting chemotaxis protein YoaH | 150 | 23 | 2.67 |
| SOV_37750 | Activator of (R)-2-hydroxyglutaryl-CoA dehydratase | 6409 | 1007 | 2.66 |
| SOV_18390 | Flagellar hook-length control protein FliK | 7991 | 1260 | 2.66 |
| SOV_30410 | Linear gramicidin synthase subunit D | 22592 | 7811 | 2.65 |
| SOV_23560 | multifunctional acyl-CoA thioesterase I and protease I and lysophospholipase L1 | 607 | 95 | 2.65 |
| SOV_46020 | Putative sporulation-specific glycosylase YdhD | 214 | 32 | 2.64 |
| SOV_29670 | Glutathione transport system permease protein GsiC | 1382 | 211 | 2.64 |
| SOV_08340 | TPR repeat-containing protein YrrB | 86 | 13 | 2.64 |
| SOV_38550 | Membrane transport protein | 146 | 23 | 2.63 |
| SOV_30730 | Multidrug resistance protein 3 | 55 | 8 | 2.63 |
| SOV_19560 | quinol dehydrogenase membrane component | 537 | 86 | 2.62 |
| SOV_21990 | Phosphopentomutase | 1532 | 249 | 2.61 |
| SOV_13020 | Ferrous iron transport protein B | 87 | 14 | 2.60 |
| SOV_14660 | Methylmalonyl-CoA carboxyltransferase 12S subunit | 16134 | 2652 | 2.60 |
| SOV_39950 | Cyclic di-GMP phosphodiesterase response regulator RpfG | 1547 | 250 | 2.60 |
| SOV_39920 | FlgN protein | 1181 | 193 | 2.60 |
| SOV_28070 | Bifunctional hemolysin/adenylate cyclase precursor | 17 | 2 | 2.59 |
| SOV_13790 | cell division inhibitor MinD | 19346 | 15941 | 2.58 |
| SOV_08330 | TPR repeat-containing protein YrrB | 64 | 10 | 2.56 |
| SOV_21840 | 1-deoxy-D-xylulose-5-phosphate synthase | 18811 | 887 | 2.54 |
| SOV_11290 | Aminopeptidase PepS | 1020 | 175 | 2.53 |
| SOV_21370 | AI-2 transport protein TqsA | 9354 | 596 | 2.53 |
| SOV_32920 | Glutamate synthase [NADPH] large chain precursor | 26 | 4 | 2.53 |
| SOV_29590 | Type II secretion system protein D precursor | 31 | 5 | 2.53 |
| SOV_44930 | Coenzyme F420:L-glutamate ligase | 338 | 58 | 2.52 |
| SOV_24390 | Putative L,D-transpeptidase YkuD | 428 | 74 | 2.52 |
| SOV_36550 | Formate-tetrahydrofolate ligase | 52484 | 8996 | 2.52 |
| SOV_51400 | Spore coat associated protein JA (CotJA) | 9 | 1 | 2.52 |
| SOV_11140 | Putative permease YicO | 6720 | 1162 | 2.51 |
| SOV_38580 | Low affinity potassium transport system protein kup | 323 | 56 | 2.50 |
| SOV_26730 | Riboflavin biosynthesis protein RibBA | 1494 | 261 | 2.49 |
| SOV_15030 | putative nicotinate-nucleotide adenylyltransferase | 969 | 171 | 2.49 |
| SOV_16430 | Phosphoserine phosphatase 1 | 1624 | 287 | 2.48 |
| SOV_00560 | CorA-like Mg2+ transporter protein | 8563 | 483 | 2.47 |
| SOV_08980 | UDP-N-acetylgalactosamine-undecaprenyl-phosphate N-acetylgalactosaminephosphotransferase | 2467 | 447 | 2.46 |
| SOV_50610 | putative manganese catalase | 47 | 8 | 2.46 |
| SOV_21870 | 16S rRNA m(4)C1402 methyltransferase | 1874 | 339 | 2.46 |
| SOV_34480 | HTH-type transcriptional regulator GltR | 148 | 26 | 2.46 |
| SOV_31050 | Stage V sporulation protein AD | 12 | 1 | 2.45 |
| SOV_19590 | flavodoxin | 79 | 14 | 2.45 |
| SOV_35420 | Hydroxylamine reductase Hcp3 | 9 | 1 | 2.43 |
| SOV_42090 | Spore germination protein B1 | 41 | 7 | 2.43 |
| SOV_32580 | Carbohydrate diacid regulator | 642 | 117 | 2.43 |
| SOV_24280 | VWA domain containing CoxE-like protein | 6558 | 1220 | 2.42 |
| SOV_51760 | Serine/threonine transporter SstT | 2680 | 498 | 2.42 |
| SOV_51880 | Periplasmic binding protein | 30 | 5 | 2.42 |
| SOV_31400 | Hexuronate transporter | 482 | 90 | 2.41 |
| SOV_30190 | Glutathione transport system permease protein GsiD | 67 | 11 | 2.40 |
| SOV_29230 | putative HTH-type transcriptional regulator YttP | 140 | 25 | 2.38 |
| SOV_10860 | Undecaprenyl phosphate-alpha-4-amino-4-deoxy-L-arabinose arabinosyl transferase | 50 | 8 | 2.38 |
| SOV_33570 | Apolipoprotein N-acyltransferase | 775 | 149 | 2.37 |
| SOV_28450 | Octanoyltransferase LipM | 107 | 20 | 2.37 |
| SOV_52610 | Sensor histidine kinase YpdA | 443 | 84 | 2.37 |
| SOV_20750 | HTH-type transcriptional activator CmpR | 65 | 11 | 2.37 |
| SOV_51960 | phosphoenolpyruvate synthase | 450 | 86 | 2.37 |
| SOV_33750 | Phenyloxazoline synthase MbtB | 252 | 48 | 2.36 |
| SOV_40960 | Chromosome partition protein Smc | 5339 | 1040 | 2.35 |
| SOV_01980 | Methionine-tRNA ligase | 14631 | 2872 | 2.35 |
| SOV_30960 | Phytochrome-like protein cph2 | 314 | 61 | 2.35 |
| SOV_19400 | putative xanthine dehydrogenase subunit A | 1200 | 235 | 2.34 |
| SOV_15420 | ATP-dependent helicase HepA | 2970 | 586 | 2.34 |
| SOV_28330 | Polyketide synthase PksN | 327 | 64 | 2.33 |
| SOV_26420 | Peptidoglycan-N-acetylglucosamine deacetylase | 687 | 135 | 2.33 |
| SOV_21390 | Oxygen-independent coproporphyrinogen-III oxidase 2 | 21952 | 15732 | 2.33 |
| SOV_01490 | Flavoredoxin | 12628 | 759 | 2.31 |
| SOV_38720 | Pentapeptide repeats (8 copies) | 60 | 12 | 2.30 |
| SOV_14060 | putative GTP-binding protein EngB | 6531 | 1319 | 2.30 |
| SOV_41980 | Serine/threonine-protein kinase PknB | 680 | 138 | 2.30 |
| SOV_44230 | Anaerobic dimethyl sulfoxide reductase chain B | 21 | 3 | 2.29 |
| SOV_51270 | DNA polymerase III PolC-type | 42 | 8 | 2.29 |
| SOV_27360 | tRNA (uracil-5-)-methyltransferase Gid | 18 | 3 | 2.29 |
| SOV_06170 | Sodium/glutamate symport carrier protein | 39 | 7 | 2.28 |
| SOV_20690 | Cell wall-binding protein YocH precursor | 16958 | 3476 | 2.28 |
| SOV_36450 | Chemotaxis protein CheY | 559 | 115 | 2.27 |
| SOV_37040 | Bacterial regulatory protein, tetR family | 19 | 3 | 2.27 |
| SOV_36390 | HTH-type transcriptional activator CmpR | 8267 | 335 | 2.26 |
| SOV_15930 | Heme/hemopexin transporter protein HuxB precursor | 66 | 13 | 2.26 |
| SOV_37940 | Spore germination protein B3 precursor | 42 | 8 | 2.26 |
| SOV_09440 | FMN-dependent NADH-azoreductase 2 | 892 | 186 | 2.25 |
| SOV_30200 | Oligopeptide transport ATP-binding protein OppD | 116 | 23 | 2.25 |
| SOV_05550 | Inner membrane ABC transporter ATP-binding protein YddA | 213 | 44 | 2.24 |
| SOV_42560 | Cyclic di-GMP phosphodiesterase Gmr | 96 | 19 | 2.24 |
| SOV_30040 | Tyrocidine synthase 3 | 315 | 66 | 2.24 |
| SOV_00460 | Cyclic di-GMP phosphodiesterase response regulator RpfG | 636 | 133 | 2.24 |
| SOV_03120 | N-acetyltransferase YodP | 8918 | 1879 | 2.23 |
| SOV_14890 | Rod shape-determining protein RodA | 930 | 194 | 2.23 |
| SOV_14050 | Lon protease 1 | 30988 | 5052 | 2.22 |
| SOV_30660 | Putative multidrug export ATP-binding/permease protein | 43 | 8 | 2.22 |
| SOV_46430 | NADP-reducing hydrogenase subunit HndC | 1026 | 219 | 2.21 |
| SOV_28760 | NADPH-dependent FMN reductase | 135 | 28 | 2.21 |
| SOV_42110 | Spore germination protein YndE | 21 | 3 | 2.21 |
| SOV_22020 | Anti-sigma F factor antagonist | 146 | 31 | 2.20 |
| SOV_00100 | PrkA AAA domain protein | 26 | 5 | 2.20 |
| SOV_27480 | Fosmidomycin resistance protein | 15967 | 230 | 2.20 |
| SOV_28030 | Hemolysin secretion protein D, chromosomal | 101 | 22 | 2.20 |
| SOV_12770 | Cyclic nucleotide-binding domain protein | 30 | 6 | 2.19 |
| SOV_40670 | Glucose-1-phosphate thymidylyltransferase 1 | 3370 | 738 | 2.18 |
| SOV_12650 | putative transport protein YifK | 96 | 20 | 2.17 |
| SOV_03100 | Glutamine transport ATP-binding protein GlnQ | 16257 | 1617 | 2.16 |
| SOV_00470 | Chaperone protein ClpB 1 | 523406 | 116152 | 2.16 |
| SOV_33250 | putative manganese catalase | 22 | 4 | 2.16 |
| SOV_24170 | Glyoxylate/hydroxypyruvate reductase B | 1002 | 224 | 2.15 |
| SOV_51770 | Serine/threonine transporter SstT | 1533 | 342 | 2.15 |
| SOV_42520 | Gliding motility regulatory protein | 2964 | 660 | 2.15 |
| SOV_11470 | Flavodoxin domain protein | 29892 | 278 | 2.14 |
| SOV_30970 | Alkaline phosphatase 4 precursor | 708 | 160 | 2.14 |
| SOV_20880 | Translation initiation factor IF-3 | 19795 | 2031 | 2.13 |
| SOV_33840 | Regulatory protein PchR | 19 | 3 | 2.13 |
| SOV_22000 | Pyrimidine-nucleoside phosphorylase | 1744 | 398 | 2.13 |
| SOV_33860 | Colicin I receptor precursor | 24 | 4 | 2.12 |
| SOV_47040 | NMT1/THI5 like protein | 1899 | 434 | 2.11 |
| SOV_10810 | Phosphomethylpyrimidine synthase | 14810 | 3423 | 2.10 |
| SOV_30180 | Dipeptide transport system permease protein DppB | 110 | 23 | 2.10 |
| SOV_08230 | Colicin I receptor precursor | 17 | 3 | 2.10 |
| SOV_15770 | PglZ domain protein | 17769 | 867 | 2.10 |
| SOV_36320 | Glutamyl-tRNA(Gln) amidotransferase subunit A | 12032 | 2795 | 2.09 |
| SOV_25060 | Periplasmic [NiFeSe] hydrogenase small subunit precursor | 21 | 4 | 2.09 |
| SOV_11240 | 26 kDa periplasmic immunogenic protein precursor | 1654 | 387 | 2.09 |
| SOV_51230 | Putative peptidoglycan binding domain protein | 174 | 39 | 2.09 |
| SOV_08090 | Bicarbonate transport system permease protein CmpB, putative nitrate/nitrite transport system | 21 | 4 | 2.09 |
| SOV_43470 | Colicin I receptor precursor | 2378 | 458 | 2.08 |
| SOV_47270 | Arginine utilization regulatory protein RocR | 1465 | 344 | 2.08 |
| SOV_44370 | Signal peptidase I S | 1640 | 387 | 2.08 |
| SOV_30670 | putative ABC transporter ATP-binding protein | 32 | 6 | 2.07 |
| SOV_42690 | Response regulator PleD | 15856 | 306 | 2.07 |
| SOV_21080 | Penicillin-binding protein 1F | 10512 | 1047 | 2.07 |
| SOV_49430 | ATP-dependent DNA helicase DinG | 5006 | 1193 | 2.06 |
| SOV_41060 | Tetraacyldisaccharide 4'-kinase | 4147 | 991 | 2.06 |
| SOV_06020 | Isocitrate dehydrogenase [NADP] | 1994 | 478 | 2.05 |
| SOV_00390 | Carboxymethylenebutenolidase | 178 | 42 | 2.05 |
| SOV_36230 | DNA-3-methyladenine glycosylase 2 | 1861 | 447 | 2.04 |
| SOV_03020 | Phosphate import ATP-binding protein PstB 3 | 233 | 56 | 2.03 |
| SOV_23790 | DNA-binding transcriptional activator FeaR | 64 | 15 | 2.03 |
| SOV_42540 | Nitrogen regulation protein NR(I) | 1397 | 334 | 2.03 |
| SOV_18680 | 30S ribosomal protein S2 | 19441 | 4730 | 2.03 |
| SOV_40810 | General stress protein A | 768 | 186 | 2.03 |
| SOV_24620 | Sensor histidine kinase YpdA | 4845 | 1203 | 2.01 |
| SOV_09140 | Peptidoglycan-N-acetylmuramic acid deacetylase PdaA precursor | 2727 | 671 | 2.00 |
| SOV_07080 | Demethylrebeccamycin-D-glucose O-methyltransferase | 6567 | 1631 | 2.00 |
| SOV_03940 | DNA-directed RNA polymerase subunit beta' | 98740 | 24658 | 2.00 |
| SOV_41070 | 3-deoxy-D-manno-octulosonic acid transferase | 6120 | 1533 | 2.00 |

^1^Mean values of normalized read counts of cells grown on fructose + nitrate and cells grown on fructose

**Table S2. The most downregulated protein-coding genes of *S. ovata* during growth in the presence of nitrate.**

| Gene | Annotation | Substrate | | Log2-fold change |
| --- | --- | --- | --- | --- |
|  |  | Fructose  + nitrate^1^ | Fructose^1^ |  |
| SOV_07570 | Coenzyme PQQ synthesis protein D | 0 | 47 | -10.33 |
| SOV_29640 | putative RNA-binding protein | 9 | 5304 | -9.16 |
| SOV_46870 | Glycine betaine/carnitine transport ATP-binding protein GbuA | 1 | 644 | -9.14 |
| SOV_45260 | Sec-independent protein translocase protein TatAd | 39 | 5339 | -7.09 |
| SOV_25400 | cobaltochelatase subunit CobN | 4 | 489 | -6.93 |
| SOV_11640 | Transposase IS200 like protein | 0 | 54 | -6.83 |
| SOV_01810 | Flp/Fap pilin component | 9 | 762 | -6.45 |
| SOV_43790 | 3-ketoacyl-(acyl-carrier-protein) reductase | 4 | 349 | -6.39 |
| SOV_22800 | Spo0E like sporulation regulatory protein | 0 | 15 | -6.35 |
| SOV_49640 | chaperone protein DnaJ | 9 | 704 | -6.31 |
| SOV_00670 | Spo0E like sporulation regulatory protein | 15 | 1066 | -6.15 |
| SOV_16540 | FeoA domain protein | 4 | 283 | -6.15 |
| SOV_26550 | 50S ribosomal protein L28 | 248 | 14534 | -5.87 |
| SOV_53210 | Putative membrane protein insertion efficiency factor | 143 | 20798 | -5.85 |
| SOV_02590 | 2-oxoglutarate-acceptor oxidoreductase subunit OorD | 18 | 1051 | -5.84 |
| SOV_02520 | Ferredoxin | 33 | 1635 | -5.59 |
| SOV_33060 | LexA repressor | 14 | 653 | -5.56 |
| SOV_47960 | transcriptional repressor DicA | 3 | 145 | -5.56 |
| SOV_20730 | Phd_YefM | 22 | 15881 | -5.43 |
| SOV_39260 | High molecular weight rubredoxin | 34 | 5732 | -5.39 |
| SOV_46910 | sulfur transfer protein SirA | 31 | 15633 | -5.37 |
| SOV_01450 | acid-soluble spore protein H | 7 | 288 | -5.33 |
| SOV_39020 | preprotein translocase subunit SecG | 500 | 19698 | -5.29 |
| SOV_51740 | Putative reactive intermediate deaminase TdcF | 10 | 373 | -5.24 |
| SOV_41410 | Stage III sporulation protein D | 0 | 14 | -5.15 |
| SOV_31800 | Molybdenum-pterin-binding protein 2 | 217 | 7663 | -5.14 |
| SOV_21680 | Stage III sporulation protein AC/AD protein family protein | 0 | 5 | -5.12 |
| SOV_46710 | Glycine betaine transporter OpuD | 32 | 1240 | -5.12 |
| SOV_51840 | Methylaspartate mutase S chain | 4 | 128 | -5.11 |
| SOV_48030 | DNA-binding transcriptional regulator SoxS | 1 | 47 | -5.00 |
| SOV_48080 | FeoA domain protein | 101 | 3198 | -4.97 |
| SOV_24010 | Zinc ribbon domain protein | 85 | 2633 | -4.94 |
| SOV_13670 | Copper-sensing transcriptional repressor CsoR | 32 | 942 | -4.87 |
| SOV_18430 | Flagellar protein (FlbD) | 94 | 2709 | -4.85 |
| SOV_19880 | 30S ribosomal protein S21 | 4751 | 37253 | -4.84 |
| SOV_38500 | Sec-independent protein translocase protein TatAy | 16 | 452 | -4.82 |
| SOV_49650 | Spo0E like sporulation regulatory protein | 14 | 408 | -4.80 |
| SOV_17740 | Stage V sporulation protein S | 179 | 4762 | -4.73 |
| SOV_40510 | Ferredoxin-2 | 44 | 1160 | -4.72 |
| SOV_37170 | Spo0E like sporulation regulatory protein | 5 | 137 | -4.68 |
| SOV_42820 | CRISPR-associated endoribonuclease Cas2 | 16 | 418 | -4.68 |
| SOV_45300 | Putative methyl-accepting chemotaxis protein YoaH | 1 | 26 | -4.64 |
| SOV_10990 | photosystem I subunit VII | 6 | 146 | -4.62 |
| SOV_26920 | Copper chaperone CopZ | 137 | 20480 | -4.62 |
| SOV_06140 | Copper chaperone CopZ | 92 | 2210 | -4.57 |
| SOV_04210 | 50S ribosomal protein L30 | 320 | 7344 | -4.52 |
| SOV_03860 | preprotein translocase subunit SecE | 1131 | 25916 | -4.51 |
| SOV_23070 | Nitrogen fixation protein of unknown function | 58 | 1319 | -4.49 |
| SOV_32300 | Methionine synthase | 6 | 149 | -4.49 |
| SOV_04160 | 30S ribosomal protein S14 | 541 | 12119 | -4.48 |
| SOV_32280 | Trimethylamine methyltransferase (MTTB) | 11 | 258 | -4.45 |
| SOV_39510 | Cold shock protein CspB | 3776 | 82054 | -4.44 |
| SOV_18730 | Ferredoxin | 1767 | 37185 | -4.39 |
| SOV_05480 | Copper chaperone CopZ | 1 | 19 | -4.38 |
| SOV_23530 | TSCPD domain protein | 55 | 1119 | -4.33 |
| SOV_26950 | DNA-directed RNA polymerase subunit omega | 91 | 1821 | -4.31 |
| SOV_41540 | Putative F0F1-ATPase subunit (ATPase_gene1) | 264 | 5029 | -4.25 |
| SOV_52790 | 30S ribosomal protein S18 | 2672 | 50854 | -4.25 |
| SOV_48440 | Phosphocarrier protein NPr | 510 | 21680 | -4.24 |
| SOV_09320 | Transcriptional activator protein CzcR | 3 | 71 | -4.24 |
| SOV_32100 | DNA polymerase IV | 6 | 101 | -4.15 |
| SOV_06120 | Copper-sensing transcriptional repressor CsoR | 10 | 179 | -4.12 |
| SOV_21810 | Exodeoxyribonuclease 7 small subunit | 41 | 724 | -4.11 |
| SOV_46760 | Methionine synthase | 176 | 9200 | -4.08 |
| SOV_46820 | 5-methyltetrahydrofolate:corrinoid/iron-sulfur protein co-methyltransferase | 81 | 1393 | -4.06 |
| SOV_09480 | CHASE4 domain protein | 2 | 47 | -4.05 |
| SOV_39990 | YvrJ protein family protein | 0 | 3 | -4.04 |
| SOV_35260 | Transposase | 13 | 222 | -4.03 |
| SOV_51410 | DNA-binding transcriptional activator PspC | 13 | 220 | -4.01 |
| SOV_49240 | Sulfite exporter TauE/SafE | 9 | 149 | -4.00 |
| SOV_14150 | Sec-independent protein translocase protein TatAd | 237 | 3764 | -3.98 |
| SOV_01970 | Transition state regulatory protein AbrB | 692 | 10843 | -3.97 |
| SOV_16090 | Late competence development protein ComFB | 150 | 5913 | -3.94 |
| SOV_35120 | flagellar motor switch protein G | 2 | 38 | -3.94 |
| SOV_23040 | Phage Tail Collar Domain protein | 92 | 1420 | -3.94 |
| SOV_19730 | 30S ribosomal protein S20 | 6662 | 98254 | -3.88 |
| SOV_14880 | cell division topological specificity factor MinE | 443 | 16315 | -3.86 |
| SOV_04270 | Translation initiation factor IF-1 | 590 | 8540 | -3.85 |
| SOV_36370 | SirA-like protein | 501 | 7267 | -3.85 |
| SOV_32530 | Pyruvate kinase | 3 | 45 | -3.83 |
| SOV_11250 | YmaF family protein | 1 | 21 | -3.82 |
| SOV_18990 | PRC-barrel domain protein | 68 | 964 | -3.81 |
| SOV_46660 | Glycine/sarcosine/betaine reductase complex component A1 | 154 | 2170 | -3.79 |
| SOV_39870 | Carbon storage regulator | 45 | 619 | -3.77 |
| SOV_53230 | 50S ribosomal protein L34 | 728 | 22110 | -3.76 |
| SOV_44770 | Acetylene hydratase | 0 | 7 | -3.71 |
| SOV_32290 | Trimethylamine methyltransferase (MTTB) | 14 | 195 | -3.71 |
| SOV_06600 | Transposase | 42 | 536 | -3.67 |
| SOV_50620 | Helix-turn-helix domain protein | 6 | 79 | -3.67 |
| SOV_10100 | 10 kDa chaperonin | 4892 | 62483 | -3.66 |
| SOV_15970 | Transition state regulatory protein AbrB | 460 | 22788 | -3.65 |
| SOV_51920 | putative ABC transporter ATP-binding protein YxlF | 0 | 6 | -3.64 |
| SOV_29460 | Helix-turn-helix domain protein | 20 | 252 | -3.64 |
| SOV_15850 | RNA polymerase sigma-35 factor precursor | 26 | 326 | -3.63 |
| SOV_27730 | Stage V sporulation protein S | 506 | 6105 | -3.59 |
| SOV_06670 | Putative zinc ribbon domain protein | 28 | 340 | -3.58 |
| SOV_41690 | 50S ribosomal protein L31 | 1803 | 21302 | -3.56 |
| SOV_04110 | 50S ribosomal protein L29 | 1332 | 15166 | -3.50 |
| SOV_46720 | Prolyl-tRNA editing protein ProX | 98 | 1230 | -3.50 |
| SOV_48410 | Multidrug-efflux transporter 1 regulator | 2 | 26 | -3.48 |
| SOV_19210 | ThiS family protein | 44 | 499 | -3.47 |
| SOV_33600 | RNA-binding protein Hfq | 17 | 180 | -3.40 |
| SOV_34740 | Plasmid stabilization system protein | 41 | 436 | -3.40 |
| SOV_27620 | RNA-binding protein Hfq | 231 | 2442 | -3.39 |
| SOV_17690 | Acyl carrier protein | 2518 | 26364 | -3.38 |
| SOV_33200 | Calcium-transporting ATPase | 0 | 4 | -3.38 |
| SOV_18510 | Flagellar biosynthetic protein FliQ | 241 | 2416 | -3.32 |
| SOV_35070 | RNA-binding protein Hfq | 13 | 137 | -3.32 |
| SOV_33970 | Transposase | 4 | 43 | -3.31 |
| SOV_46790 | Glycine reductase complex component B subunits alpha and beta | 529 | 5765 | -3.30 |
| SOV_45970 | putative nicotinate-nucleotide pyrophosphorylase [carboxylating] | 40 | 407 | -3.30 |
| SOV_00130 | Butyrate kinase 2 | 0 | 5 | -3.27 |
| SOV_44670 | Sulfur carrier protein ThiS | 6 | 59 | -3.26 |
| SOV_49150 | antitoxin HipB | 25 | 236 | -3.25 |
| SOV_09960 | Antitoxin EndoAI | 420 | 4000 | -3.25 |
| SOV_36530 | HTH-type transcriptional activator HxlR | 49 | 474 | -3.24 |
| SOV_41510 | ATP synthase subunit c, sodium ion specific | 10300 | 71171 | -3.22 |
| SOV_39130 | Hydrogenase isoenzymes formation protein HypC | 110 | 1030 | -3.22 |
| SOV_20980 | Cell division protein ZapA | 58 | 544 | -3.21 |
| SOV_23680 | Chemotaxis protein CheY | 50 | 460 | -3.19 |
| SOV_46750 | Trimethylamine methyltransferase (MTTB) | 742 | 7016 | -3.19 |
| SOV_33190 | Calcium-transporting ATPase 1 | 0 | 8 | -3.11 |
| SOV_03840 | 50S ribosomal protein L33 1 | 788 | 6812 | -3.10 |
| SOV_46770 | Glycine reductase complex component B subunit gamma | 552 | 17553 | -3.09 |
| SOV_33620 | flagellar motor switch protein G | 12 | 109 | -3.09 |
| SOV_27760 | recombination regulator RecX | 210 | 1758 | -3.05 |
| SOV_44970 | Phosphocarrier protein HPr | 106 | 886 | -3.05 |
| SOV_32270 | Trimethylamine methyltransferase (MTTB) | 48 | 407 | -3.04 |
| SOV_22090 | SpoVA protein | 0 | 6 | -3.02 |
| SOV_16900 | Transglycosylase associated protein | 3 | 29 | -3.02 |
| SOV_49230 | Sulfite exporter TauE/SafE | 18 | 152 | -3.01 |
| SOV_52390 | HTH-type transcriptional regulator SinR | 34 | 284 | -3.01 |
| SOV_12120 | leucine/isoleucine/valine transporter permease subunit | 3 | 30 | -3.00 |
| SOV_07200 | Anti-sigma F factor | 109 | 908 | -3.00 |
| SOV_14130 | NADH dehydrogenase subunit I | 4 | 33 | -2.98 |
| SOV_06730 | Transcriptional regulatory protein AfsQ1 | 2 | 21 | -2.98 |
| SOV_38230 | SpoVT / AbrB like domain protein | 28 | 226 | -2.97 |
| SOV_31650 | CTP pyrophosphohydrolase | 51 | 406 | -2.97 |
| SOV_46730 | Acetophenone carboxylase gamma subunit | 842 | 20125 | -2.92 |
| SOV_18910 | 30S ribosomal protein S15 | 2766 | 29648 | -2.89 |
| SOV_15040 | RNA recognition motif | 43 | 313 | -2.87 |
| SOV_46810 | Vitamin B12 dependent methionine synthase, activation domain | 5 | 41 | -2.87 |
| SOV_51650 | anaerobic ribonucleoside triphosphate reductase | 406 | 10481 | -2.86 |
| SOV_04120 | 30S ribosomal protein S17 | 1342 | 21708 | -2.85 |
| SOV_03230 | Cell division protein FtsL | 363 | 19057 | -2.84 |
| SOV_46640 | Glycine/sarcosine/betaine reductase complex component C subunit alpha | 224 | 5191 | -2.83 |
| SOV_15200 | Integral membrane protein TerC family protein | 7 | 69 | -2.81 |
| SOV_06770 | Macrolide export ATP-binding/permease protein MacB | 1 | 9 | -2.80 |
| SOV_05570 | Helix-turn-helix domain protein | 2 | 16 | -2.79 |
| SOV_12330 | ThiS family protein | 0 | 6 | -2.79 |
| SOV_52010 | Vitamin B12-binding protein precursor | 55 | 380 | -2.75 |
| SOV_22810 | Phage terminase small subunit | 6 | 45 | -2.70 |
| SOV_36780 | Trp repressor protein | 77 | 507 | -2.70 |
| SOV_52810 | 30S ribosomal protein S6 | 3159 | 19940 | -2.65 |
| SOV_05440 | CAAX amino terminal protease self- immunity | 3 | 27 | -2.65 |
| SOV_46650 | Glycine/sarcosine/betaine reductase complex component C subunit beta | 287 | 1822 | -2.64 |
| SOV_50340 | Transcriptional regulatory protein DegU | 480 | 7897 | -2.62 |
| SOV_14690 | Glutaconyl-CoA decarboxylase subunit beta | 328 | 2005 | -2.60 |
| SOV_46690 | Thioredoxin reductase | 199 | 1227 | -2.60 |
| SOV_27930 | Chemotaxis protein CheY | 139 | 848 | -2.59 |
| SOV_01150 | Plasmid pRiA4b ORF-3-like protein | 46 | 277 | -2.58 |
| SOV_09780 | Transposase IS200 like protein | 60 | 363 | -2.57 |
| SOV_36080 | Putative sensory transducer protein YfmS | 403 | 10475 | -2.55 |
| SOV_03000 | DNA-binding protein HU | 3169 | 18710 | -2.55 |
| SOV_17040 | HTH-type transcriptional regulator ImmR | 3 | 22 | -2.53 |
| SOV_36070 | Chemotaxis protein CheW | 153 | 925 | -2.51 |
| SOV_17910 | L,D-transpeptidase catalytic domain | 227 | 1308 | -2.50 |
| SOV_12020 | HicB family protein | 546 | 3116 | -2.50 |
| SOV_40300 | Nucleotidyltransferase domain protein | 66 | 373 | -2.49 |
| SOV_22420 | Helix-turn-helix domain protein | 3 | 21 | -2.48 |
| SOV_42660 | HTH-type transcriptional repressor RghR | 123 | 694 | -2.48 |
| SOV_46680 | Thioredoxin | 30 | 182 | -2.47 |
| SOV_07350 | Putative glycosyltransferase EpsE | 1 | 11 | -2.47 |
| SOV_46740 | Trimethylamine methyltransferase (MTTB) | 1016 | 5878 | -2.47 |
| SOV_04070 | 30S ribosomal protein S19 | 1878 | 10424 | -2.46 |
| SOV_15070 | Ribosomal silencing factor RsfS | 252 | 1401 | -2.46 |
| SOV_51130 | Phosphoribosyl-ATP pyrophosphatase | 27 | 151 | -2.45 |
| SOV_15810 | RNA polymerase sigma-35 factor precursor | 64 | 355 | -2.44 |
| SOV_46700 | Glycine betaine transporter OpuD | 267 | 1538 | -2.44 |
| SOV_30080 | Polyketide biosynthesis malonyl CoA-acyl carrier protein transacylase BaeC | 1 | 9 | -2.42 |
| SOV_23360 | putative reductase | 71 | 384 | -2.42 |
| SOV_44910 | Acireductone dioxygenase | 53 | 292 | -2.41 |
| SOV_47780 | Riboflavin transporter RibU | 274 | 10696 | -2.37 |
| SOV_01510 | Nitrogen regulatory protein P-II | 9 | 47 | -2.36 |
| SOV_49810 | Transposase IS200 like protein | 5 | 29 | -2.34 |
| SOV_17240 | Single-stranded DNA-binding protein ssb | 5 | 29 | -2.34 |
| SOV_03970 | Ribosome-associated protein L7Ae-like protein | 14511 | 5593 | -2.30 |
| SOV_20290 | Undecaprenol kinase | 170 | 843 | -2.30 |
| SOV_17620 | 50S ribosomal protein L32 | 1268 | 6219 | -2.29 |
| SOV_23430 | Amino-acid acetyltransferase | 155 | 766 | -2.28 |
| SOV_52690 | PTS-dependent dihydroxyacetone kinase, phosphotransferase subunit DhaM | 273 | 1334 | -2.28 |
| SOV_06010 | Acetamidase/Formamidase family protein | 12 | 61 | -2.26 |
| SOV_31520 | putative M18 family aminopeptidase 1 | 261 | 1237 | -2.23 |
| SOV_31150 | LysM domain/BON superfamily protein | 11 | 53 | -2.23 |
| SOV_32170 | Methionine synthase | 700 | 3258 | -2.21 |
| SOV_04550 | VRR-NUC domain protein | 12 | 61 | -2.21 |
| SOV_11690 | Transposase IS200 like protein | 71 | 332 | -2.20 |
| SOV_28710 | HEPN domain protein | 11 | 55 | -2.20 |
| SOV_36090 | Formiminotransferase-cyclodeaminase | 5 | 27 | -2.19 |
| SOV_39590 | Nucleotidyltransferase domain protein | 158 | 723 | -2.17 |
| SOV_10050 | Small, acid-soluble spore protein D | 1 | 10 | -2.16 |
| SOV_10320 | HTH-type transcriptional regulator AdhR | 37 | 171 | -2.15 |
| SOV_27690 | Sulfite exporter TauE/SafE | 95 | 426 | -2.14 |
| SOV_10210 | putative HTH-type transcriptional regulator YvdT | 74 | 329 | -2.13 |
| SOV_47980 | Cystathionine gamma-synthase/O-acetylhomoserine (thiol)-lyase | 49 | 215 | -2.10 |
| SOV_31330 | Peptide methionine sulfoxide reductase MsrB | 67 | 296 | -2.10 |
| SOV_40320 | Phd_YefM | 85 | 366 | -2.09 |
| SOV_11910 | Citrate lyase acyl carrier protein | 16 | 75 | -2.08 |
| SOV_12990 | DGC domain protein | 726 | 8748 | -2.08 |
| SOV_33410 | flagellar motor switch protein G | 38 | 165 | -2.07 |
| SOV_51380 | Manganese containing catalase | 0 | 5 | -2.04 |
| SOV_02920 | Murein hydrolase activator NlpD precursor | 152 | 637 | -2.04 |
| SOV_48730 | SigmaK-factor processing regulatory protein BofA | 166 | 686 | -2.04 |
| SOV_45320 | Sporulation kinase A | 267 | 14718 | -2.03 |
| SOV_20890 | 50S ribosomal protein L35 | 440 | 1783 | -2.01 |
| SOV_31860 | Cytochrome c-type biogenesis protein CcmE | 55 | 229 | -2.00 |

^1^Mean values of normalized read counts of cells grown on fructose + nitrate and cells grown on fructose
